# Gut microbiota modulation enhances the immune capacity of lizards under climate warming

**DOI:** 10.1186/s40168-023-01736-2

**Published:** 2024-02-22

**Authors:** Jing Yang, Weiqiang Liu, Xingzhi Han, Xin Hao, Qibin Yao, Weiguo Du

**Affiliations:** 1 Key Laboratory of Animal Ecology and Conservation Biology, Institute of Zoology, Chinese Academy of Sciences, Beijing, 100101 China; 2 University of Chinese Academy of Sciences, Beijing, 100049 China; 3 College of Wildlife and Protected Areas, Northeast Forestry University, Harbin, 150040 China; 4 School of Tropical Agriculture and Forestry (School of Agricultural and Rural, School of Rural Revitalization), Hainan University, Danzhou, 571737 China

**Keywords:** Climate change, Intestinal microflora, Immune capacity, Microbial symbiont, Reptile, Temperature

## Abstract

**Background:**

Host-microbial interactions are expected to affect species’ adaptability to climate change but have rarely been explored in ectothermic animals. Some studies have shown that short-term warming reduced gut microbial diversity that could hamper host functional performance.

**Results:**

However, our longitudinal experiments in semi-natural conditions demonstrated that warming decreased gut microbiota diversity at 2 months, but increased diversity at 13 and 27 months in a desert lizard (*Eremias multiocellata*). Simultaneously, long-term warming significantly increased the antibacterial activity of serum, immune responses (higher expression of intestinal immune-related genes), and the concentration of short-chain fatty acids (thereby intestinal barrier and immunity) in the lizard. Fecal microbiota transplant experiments further revealed that increased diversity of gut microbiota significantly enhanced antibacterial activity and the immune response of lizards. More specifically, the enhanced immunity is likely due to the higher relative abundance of *Bacteroides* in warming lizards, given that the bacteria of *Bacteroides fragilis* regulated IFN-β expression to increase the immune response of lizards under a warming climate.

**Conclusions:**

Our study suggests that gut microbiota can help ectotherms cope with climate warming by enhancing host immune response, and highlights the importance of long-term studies on host-microbial interactions and their biological impacts.

**Graphical Abstract:**

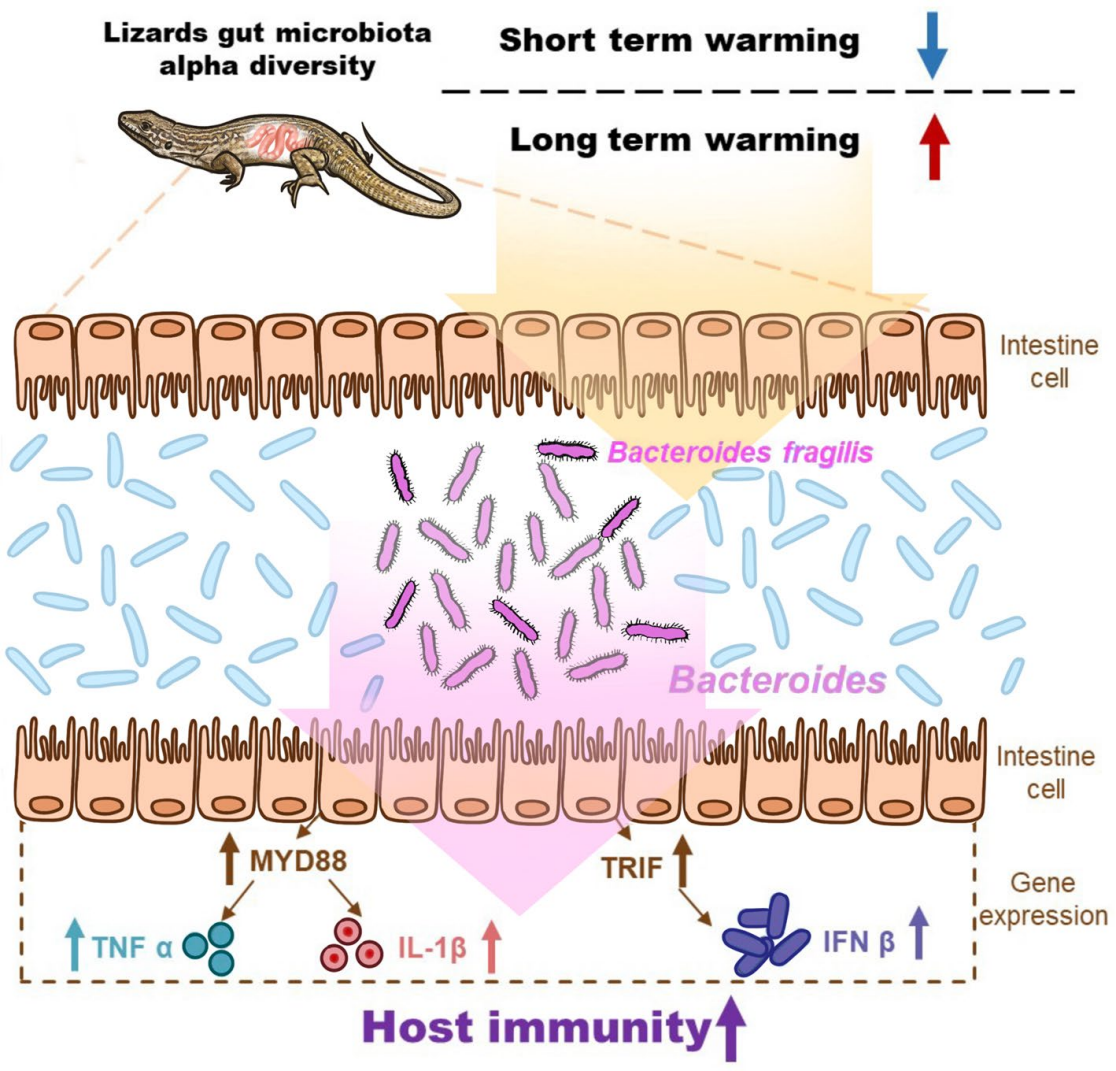

Video Abstract

**Supplementary Information:**

The online version contains supplementary material available at 10.1186/s40168-023-01736-2.

## Background

Climate change poses a serious threat to global biodiversity and viable ecosystem functioning. Threats include a decline in survival rates of many species, increased prevalence of pathogens, and increased risk of species extinctions [1–4]. Traditional studies have focused on how climate change affects the behavior, physiology, and evolution of organisms [5–9]. To date, little attention has been paid to the effects of climate change on the role of host-associated microbial communities (i.e., microbiomes), which colonize almost all organisms as symbionts [10]. However, a growing body of evidence shows that microbial symbionts may regulate the way hosts respond to their external environment [11–13]. Therefore, the way in which microbial symbiosis modulates host behavior and physiology could be crucial in determining the adaptability of hosts to climate change.

Recent research on warming experiments demonstrated that climate warming reduced gut microbial diversity in some ectotherms [14, 15]. Additionally, warming-induced loss of microbiota can reduce thermal tolerance in ectothermic hosts [15, 16]. Nonetheless, the conclusion that climate warming reduces gut microbiota diversity is drawn from the results of short-term warming experiments. How gut microbiota respond to long-term climate warming is critical for understanding the biological impact of climate change, but remains unknown. In addition, mechanisms underlying the thermal effects on the interaction between gut microbiomes and their hosts are also currently unknown. One possible mechanism is that temperature changes may alter the composition of gut microbiota, enabling hosts to adapt to climate warming by altering the immune system in ectothermic vertebrates [17, 18]. For example, the gut microbiome can modulate the host immune system by disrupting the balance between pro-inflammatory and regulatory responses, or through regulation of cytokine signaling [19–21]. However, it is still unclear whether changes in immune capacity are a direct result of temperature, microbial alteration, or both [22]. Experiments in natural conditions are needed to disentangle the interaction of temperature and microbiome and the role they play in immune function. Given that high temperatures can facilitate the colonization of pathogenic bacteria in soil, and foraging and nesting by ectotherms may in turn increase the risk of pathogen infection [23, 24], we hypothesize that alterations in the gut microbiota of ectotherms in a warming climate may enhance their immunity that resists pathogen infection.

To answer these questions, we carried out field longitudinal experiments to determine the effect of climate warming on gut microbiota and immune capacity in a viviparous desert lizard (*Eremias multiocellata*) facing relatively high rates of climate warming [25]. We also conducted fecal microbiota transplant (FMT) experiments to verify gut microbiota modulation of lizard immunity. Finally, we conducted colonization experiments to identify the key bacteria that modulate the immune capacity of hosts.

## Methods

### Warming experiments

We built open-top chambers to conduct warming experiments in the desert steppe region of Inner Mongolia, China (40.2N, 111.1E; elevation 1036 m). Each experimental chamber was 5 m in circumference and built with a 1-m-high steel plate extending 0.5 m above and 0.5 m below the ground. The warming climate chambers were covered with nets (to keep out predators) and transparent plastic film with a 0.4-m-diameter hole at the center, while the present climate chambers were only covered with nets (Fig. S1). We recorded hourly ground-surface temperatures in the chambers using thermochron iButtons (DS1921, MAXIM Integrated Products Ltd.) and relative humidity using humidity loggers (HOBO U12-012, Onset) placed in the artificial climate chamber [26]. We conducted longitudinal experiments by setting up a series of experimental populations over several years from 2018 to 2021. From May to September (the active months of lizard) of each year, the mean temperature of 29.3 ± 0.5 °C in the warming climate chambers closely mimicked the temperature of future climate (2081–2100) under SSP5-8.5 in our region, which is predicted by the BCC-CSM2-MR model in CMIP6 [27, 28]. Present climate chambers had a mean temperature of 26.4 ± 0.4 °C which closely mimicked current temperatures. The average daily maximum temperature was 38.2 °C in the warming climate chambers, and 35.3 °C in the present climate chambers. The average daily minimum temperature for both warming and present climate chambers was 9.4 °C. Relative humidity did not differ between warming climate chambers (58.5 ± 1.3%) and present climate chambers (56.0 ± 1.5). Further details of the open-top chamber system we used are described in Hao et al. [26].

Our study animal, the multiocellated racerunner (*Eremias multiocellata*), is a small viviparous lizard [44–77 mm snout-vent length (SVL)] and is abundant in our study area [26]. We randomly captured lizards from natural populations every year and placed two females and two males in the different single  chamber. A clump of *Artemisia ordosica* (the dominant vegetation in the desert steppe habitat) was planted in the middle of each chamber to provide thermal heterogeneity for behavioral thermoregulation by lizards [28]. We provided food (larvae of *Tenebrio molitor* and crickets) to lizards every 3 days. We monitored the survival of lizards in the chambers through mark-recapture in May and September every year. Survival analyses were performed using the libraries “survminer” and “survival” in R, and differences in survival rates were compared using generalized linear mixed models (GLMM) with binomial distributions.

### Analysis of environmental and lizard gut microbiota

#### Sample collection

In 2020 and 2021, we collected fecal samples from 10 wild-caught lizards, and 62 lizards that had been maintained in the chambers for 2 months (present *N* = 11; warming *N* = 11), 13 months (present *N* = 11; warming: *N* = 9), and 27 months (present *N* = 9; warming *N* = 11) to investigate the effects of climate warming on the gut microbiota of lizards. We collected fecal samples from two adult lizards in each chamber. To avoid potential contamination, we palpated the abdomen of lizards to discharge feces directly into a 2-mL sterile Eppendorf tube. Additionally, we collected soil samples from 20 chambers that had been exposed to 27 months of the warming climate treatment. To do this, we collected 3 cm of topsoil from five areas within each chamber. The soil was placed into 50-mL sterile tubes and mixed thoroughly. Following collection, samples were transported to the laboratory in a block of solid carbon dioxide and stored in a – 80 °C refrigerator for later analysis. See Table S1 in the Supplementary Materials for detailed sample information.

#### DNA extraction and 16S rRNA gene profiling

Microbial DNA from fecal samples was extracted using TIANamp Stool DNA Kit (DP328, TIANGEN, China), and DNA from soil samples was extracted using DNeasy PowerSoil Pro Kit (QIAGEN, Germany) following the manufacturer’s protocol. The concentration of extracted DNA was measured by the A260/A280 ratio on a Nanodrop 2000 (Thermo Fisher Scientific, Carlsbad, CA, USA).

The V3–V4 hypervariable regions of the 16S rRNA gene were PCR-amplified with primers (338F: ACTCCTACGGGAGGCAGCA, 806R: GGACTACHVGGGTWTCTAAT). PCR amplifications (total 25 μL) were performed using 5 μL of buffer, 1 μL of each primer, 0.25 μL of Fast pfu DNA Polymerase, 2 μL of dNTPs, and 1 μL of DNA template. PCR amplification cycles consisted of denaturation at 98 °C for 5 min, then 25 cycles of denaturation at 98 °C for 30 s, annealing at 53 °C for 30 s, and elongation at 72 °C for 45 s, with a final elongation of 5 min at 72 °C. PCR amplicons were purified using Vazyme VAHTSTM DNA Clean Beads (Vazyme, Nanjing, China) and quantified using the Quant-iT PicoGreen dsDNA Assay Kit (Invitrogen, Carlsbad, CA, USA). Purified amplicons were sequenced on the Illumina NovaSeq platform (Shanghai Personal Biotechnology Co., Ltd.) with pair-end reads of 250 bp.

#### Data analysis of 16S rRNA gene sequencing

Raw reads were imported into the QIIME2 (v2020.11.1) [29] for adapter removal, filtering, denoising, and calculation of diversity metrics. Cutadapt (v3.1) [30] was used to trim the adapters that had at least 90% base overlap between reads. The maximum error rate was 0.2, and we discarded reads in which no adapter was found. The amplicon sequence variants (ASVs) were generated by DADA2 (v1.18.0) [31]. We truncated the lengths of forward and reverse reads to 220 and 225, respectively, and filtered out pairs of reads with a forward read error rate higher than 2 and a reverse read error rate higher than 4. Since the default parameters removed too many chimeras, we adjusted the parameter ‘--p-min-fold-parent-over-abundance’ to 8 (Table S2). Our final sample size of 58,164 ASVs (we also got 25,308 environment samples and 11,500 FMT samples) was used for downstream analysis. We used MAFFT (v7.475) [32] and FastTree (v2.1.10) [33] to align sequences and construct the phylogenetic tree. The taxonomy of these features was assigned to the SILVA database (silva-138-99-nb-classifier.qza) [34] using QIIME2’s ‘qiime feature-classifier classify-sklearn’ command with default parameters. We obtained relative abundance values at different taxonomic levels using the script ‘summarize_taxa.py’ of QIIME2.

#### Time series analysis

To identify the response of bacterial communities in a warming climate, based on changes in relative abundance over time, we used the gut microbiota from 10 sympatric wild-caught lizards as the reference point of the experiment. A relative abundance score [35] was calculated using the following equation:

$$\mathrm{Score }= (1-|{\text{A}}|) \times \mathrm{ B}$$

where *A* is the Pearson correlation between the curves for a given abundance over time between the present and warming climate treatments, and *B* is the integrated numerical difference between the same curves. Thus, for a certain bacterial community, *A* tending towards 0 means its relative abundance does not correlate over time between the two climates. Since *B* describes the absolute area between the two climates over time, a high value of *B* suggests a large difference in relative abundance for a given microbial community between the two climate treatments. For example, a microbial taxonomy can be detected when it changes dramatically over time as a result of a warming climate, but not as a result of the present climate.

#### Analysis of alpha and beta diversity metrics

To assess differences in the diversity of a lizard’s gut microbiota community between present climate and warming climate groups, we performed alpha and beta diversity analysis. To normalize library size, the number of reads per sample was rarefied to 33,601 (we also rarefied to 43,520 environment samples and 30,446 FMT samples). QIIME2’s ‘qiime diversity core-metrics-phylogenetic’ command with default parameters was used to calculate alpha diversity indices and beta diversity metrics. For alpha diversity, we selected Observed features, Chao 1 index [36], Shannon’s index [37], and Faith’s PD [38] to account for species richness and evenness, and significance was evaluated by a two-sided Wilcoxon rank sum test (Table S3). For beta diversity, we used unweighted UniFrac distance to highlight the differences in species composition between individual lizard gut microbiota communities. Permutation multivariate analysis of variance (PERMANOVA) [39] was performed to reveal the effect of a warming climate on lizard gut microbiota. We used the ‘adonis2’ function in R package vegan (v2.6-2) [40] for PERMANOVA analysis with default parameters (unweighted UniFrac distance and 1000 permutations). We performed non-metric multidimensional scaling (NMDS) based on unweighted UniFrac distance using the ‘metaMDS’ function in R package vegan.

#### LDA effect size analysis

To identify biomarkers among different climate treatments, we performed linear discriminant analysis (LDA) on effect size (LEfSe) [41] based on relative abundance values at the genus level. Prior to this, we filtered out those ASVs with unclear species classification. Significance was evaluated by a two-sided Wilcoxon rank sum test. We set 1,000,000 as the normalization value. Biomarkers were identified with a *P* value < 0.05 and the LDA score cutoff was > 3.2 for fecal samples (Table S4).

#### Metagenomic function prediction

To predict the function of each ASV, we used PICRUSt2’s (v2.3.0_b) pipeline ‘pathway_pipeline.py’ with default parameters [42] to obtain the abundance data for each KEGG pathway. First, the sequence of each ASV was aligned with the SILVA database to obtain the closest 16S rRNA gene for each ASV. The copy number of the respective 16S rRNA gene for each ASV was then normalized to eliminate misestimation of gene abundance due to multiple copies of the gene. Finally, the function of metagenomics was predicted according to the Kyoto Encyclopedia of Genes and Genomes (KEGG) database [43]. To detect differences in the abundance of the KEGG pathway, we performed differential abundance analysis based on a zero-inflated log-normal model using the ‘fitFeatureModel’ function in the R package metagenomeSeq (v1.36.0) [44] with 1000 bootstraps (Table S5).

### Immunity measurement

We captured eight adult lizards that had been kept for 27 months from each treatment of the present and warming climates. Subsequently, these lizards were transported to our laboratory in Beijing for further experiments. After the lizards had been euthanized, serum and intestinal tissue were collected using sterilized tubes and scissors.

#### Histological analysis of intestinal mucosa

To investigate the health of the lizard’s intestinal mucosa, we carried out histological analysis on the samples. Ileum samples were fixed with 4% paraformaldehyde and paraffin embedded upon collection. Modified tissue blocks were cut into 4-μm slice thickness and stained with hematoxylin-eosin (HE). The target area of analysis for the tissue blocks was a ×40 image, filled with the whole field of vision. After imaging was completed, Image-Pro Plus 6.0 analysis software was used to measure the height (mm) of five intact intestinal villi and the mucosal thickness of five sites in each section. Average values were then calculated.

#### Serum antibacterial activity

To verify the immune capacity of lizards, we measured serum antibacterial activity. Whole blood samples were collected from a lizard’s eye socket with a capillary glass tube and stored at 4 °C until the serum separated. Serum was then isolated via refrigerated centrifugation at 3000 × *g* for 20 min, then frozen at −20 °C. *Escherichia coli* (BNCC133264, BeNa Culture Collection) and *Aeromonas hydrophila* (BNCC336453) were grown for 24 h at 37 °C with constant agitation (140 r/min) to reach log-phase growth, respectively. The bacterial solution was then diluted with sterile phosphate-buffered saline (PBS) until the absorbance at 680 nm was 0.20 and then diluted 100-fold to make the working solution. Five microliters of serum were mixed with 20 μL of bacterial solution and 25 μL of sterile PBS culture at 37 °C for 30 min; 20 μL of bacterial solution with 30 μL of sterile PBS was used as the control solution. Next, the serum-bacterial solution was plated on the surface of a nutrient broth agar plate and incubated at 37 °C overnight. To achieve maximum robustness of the results, two technical replicates were set for each sample. Finally, the antibacterial activity of serum was calculated using the following equation:

$$\mathrm{BKA }= (1-\mathrm{the\, mean\, number\, of\, clones\, for\, each\, sample}/\mathrm{the\, mean\, number\, of\, clones\, for\, the\, control}) \times 100\mathrm{\%}$$

#### RNA extraction and qRT-PCR gene expression analysis

To demonstrate the activation of intestinal immunity in lizards, we performed an expression analysis of intestinal immunity-associated genes. Intestinal tissue was obtained immediately after the lizards were euthanatized, and the tissues were placed in tubes and frozen promptly using liquid nitrogen. RNA of intestinal tissues was isolated using TRIzol Reagent (CWBIO). The concentration of extracted RNA was then measured using a Nanodrop 2000 (Thermo Fisher Scientific, Carlsbad, CA, USA). cDNA was prepared using HiFiScript cDNA Synthesis Kit (CW2569M, CWBIO) genes, and UltraSYBR Mixture (CW2601H, CWBIO) was used for quantitative real-time PCR by LightCycler 480 Instrument II (ROCHE, Germany). Each sample was run in triplicate, and the common wall lizard (*Podarcis muralis*) EF1A1 gene was used as the internal control. Sequences of the primers are shown in Table S6.

### Measurements of short-chain fatty acids (SCFAs)

SCFAs were extracted from fecal samples using 900 µL methanol and 100 µL 2-ethylbutyric acid (1000 μg/mL) as the internal standard. Six SCFAs (Acetate, Propionate, Butyrate, Isobutyrate, Valerate, Isovalerate) were measured via an Agilent 8890B-5977B GC/MSD (gas chromatography/mass selective detector) (Agilent, USA). An HP FFAP capillary column (30 m × 0.25 mm × 0.25 μm, Agilent J&W Scientific, Folsom, CA, USA) was used for the carrier gas with 1 μL of high-purity helium (purity not less than 99.999%). The gas chromatography column temperature was programmed to hold at 80 °C and rise to 120 °C at a rate of 40 °C/min, then rise to 200 °C at 10 °C/min, finally holding at 230 °C for 6 min. Masshunter software (v10.0.707.0, Agilent) was used for the identification and quantification of the compounds (Table S7).

### Fecal microbiota transplantation (FMT)

We carried out fecal microbiota transplant experiments to verify the gut microbiome modulation of immune capacity in lizards exposed to different climate treatments. We collected *N* = 3 and *N* = 5 adult male lizards (kept for 27 months) from different chambers of the present and warming climate treatments as donors, respectively. We dissolved fecal pellets from the two groups of donor lizards then made a supernatant with sterile PBS (add 1 mL of sterile PBS per 100 μg), and used the supernatant for FMT after it had been centrifuged at 500 × *g* for 1 min. We used 16 adult males captured from the wild as recipients (sample details and sequencing information in Tables S8 and S9) to avoid the confounding effect of female reproductive status. The recipient lizards were treated with 60 μL of composite antibiotics (containing 100 μg/mL neomycin, 50 μg/mL streptomycin, and 100 U/mL penicillin; YUANYE Bio-Technology Co., Ltd., China) every day during 7 days via intragastric gavage before FMT [45]. The recipient lizards were then allocated equally into two groups, and received 60 μL of the microbiome supernatant from the present or warming climate groups via intragastric gavage for 7 days, using syringes equipped with blunt end gavage needles, respectively. In this experiment, the recipients were housed in 6 sterilized plastic containers in a temperature-controlled room at 25.5 ± 0.1 °C. The water and sand provided in the containers were also sterilized. Fecal and tissue samples were collected after FMT for 1 week.

### Colonization with *Bacteroides fragilis*

Due to the 16S rRNA gene sequencing, distinguishing bacteria at the species level was not feasible. Thus, we used *Bacteroides fragilis* as a representative to verify the immune effect of the genus *Bacteroides* on a lizard’s gut microbiota [21]. *B. fragilis* BNCC336948 strain was cultured anaerobically at 37 °C on Columbia blood agar plates. The bacterial cells were washed with sterile PBS on the plate, then the bacterial solution was diluted to 10^8^–10^9^ colony-forming units (CFU). We depleted the microbiota of an additional *N* = 16 male lizards using the same methods as for FMT. Sixty microliters of *B. fragilis* bacteria were administered to *N* = 8 lizards by oral gavage for 7 days. The remaining eight lizards were treated with 60 μL sterile PBS. All lizards were housed in sterilized plastic containers in a temperature-controlled room for 2 weeks.

## Results

### Effects of climate warming on environmental microbiota

To detect any changes in the experimental chamber environment of lizards after 27 months, we performed microbial analysis of soil from both the warming and present climate chamber treatments. The alpha diversity of environmental microbiota was lower in the warming climate chambers than in the present climate chambers after 27 months (*P* = 2.38 × 10^−5^, Fig. S2a), and the composition of the bacterial communities within each group showed a significant separation (*P* = 0.001, Fig. S2b). At the genus level, there was a significant increase in the relative abundance of *Pseudarthrobacter* (*P* = 1.19 × 10^−5^), *Massilia* (*P* = 2.38 × 10^−5^), and *Cellulomonas* (*P* = 0.001) from the warming climate chambers (Fig. S2c). These results suggest that climate warming will reduce the microbial diversity of a lizard’s environment, but will increase the abundance of some bacteria.

### Dynamics of lizard gut microbiota under a warming climate

**Fig. 1:**
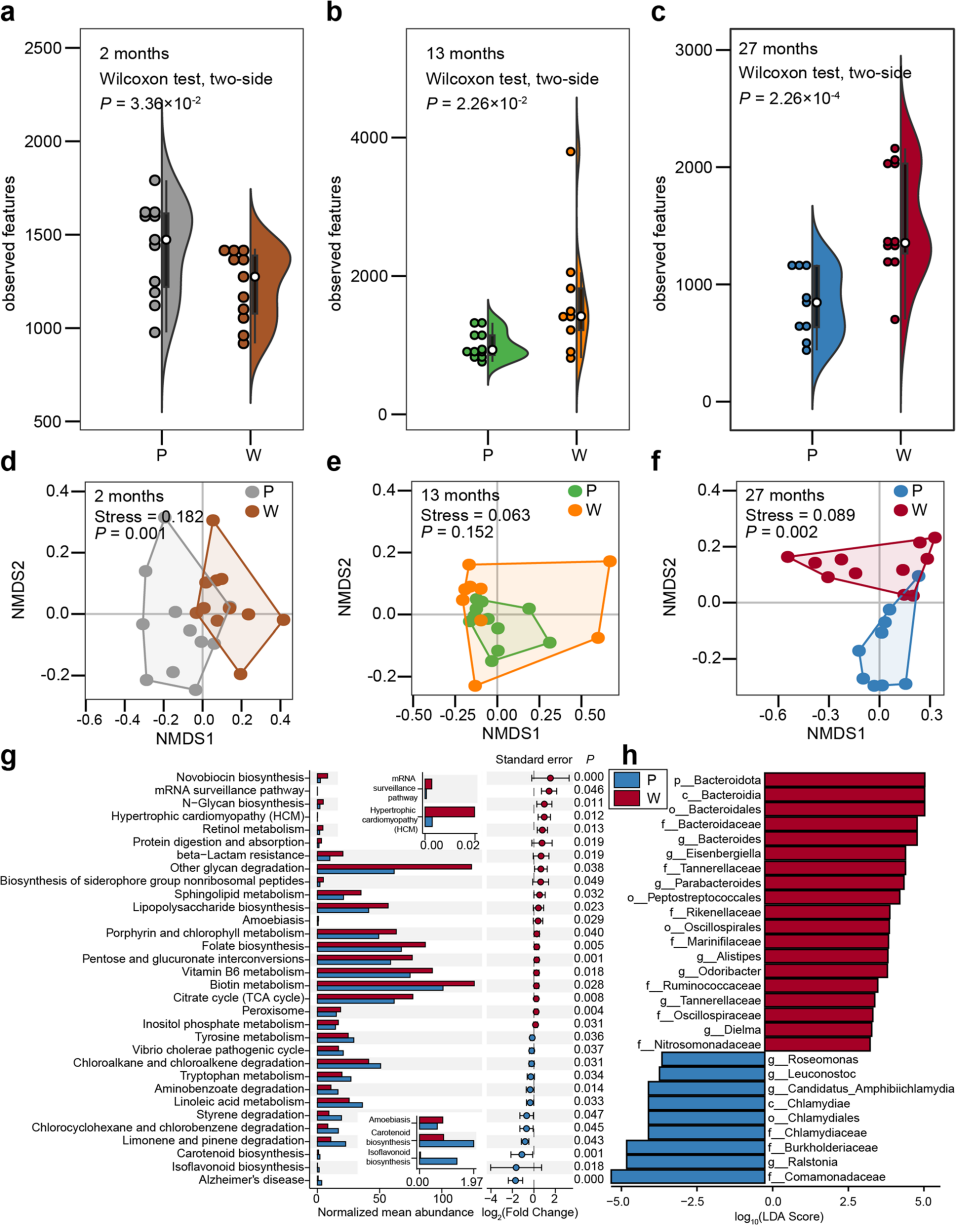
Effects of climate warming on gut microbiota of lizards. P represents present climate. W represents warming climate. The significance of warming effects was evaluated by a two-sided Wilcoxon rank sum test. The significance of non-metric multidimensional scaling (NMDS) analysis was evaluated by PERMANOVA with 1000 permutations. **a**–**c** The alpha diversity index (observed features) of lizard gut microbiota at 2 months (**a**), 13 months (**b**), and 27 months (**c**). **d**–**f** NMDS plot based on unweighted UniFrac distances of lizard gut microbiota at 2 months (**d**), 13 months (**e**), and 27 months (**f**). **g** The functional profiles of gut microbiota identified by PICRUSt2 using KEGG database in the 27-month group of lizards. **h** Biomarkers of discriminative bacteria identified by LEfSe analysis (LDA score ≥ 3.2) in the 27-month group of lizards

To explore the dynamic changes of lizard gut microbial communities in a warming climate, we performed diversity and composition analyses of bacteria in lizard feces. The alpha diversity of lizard gut microbiota decreased in the 2-month warming climate group compared to the present climate group (*P* = 3.36 × 10^−2^, Fig. 1a, Supplementary results), but increased in the 13-month (*P* = 2.26 × 10^−2^, Fig. 1b, Supplementary results) and 27-month warming climate groups (*P* = 2.26 × 10^−4^, Fig. 1c, Supplementary results). The beta diversity of lizard gut microbiota differed significantly between present and warming climate groups at 2 months (*P* = 0.001, Fig. 1d) and 27 months (*P* = 0.002, Fig. 1f), but not at 13 months (*P* = 0.152, Fig. 1e). Therefore, the diversity of gut microbiota decreased with short-term climate warming but increased over long-term climate warming.

### Identifying crucial bacteria in response to climate warming

To determine the physiological changes in lizards and identify the crucial bacteria present in response to climate warming, we performed functional prediction, LEfSe analysis, and time series analysis of gut bacteria. The results of differential KEGG metabolic pathway analysis (Fig. 1g and Fig. S3a, c) found that two metabolic pathways were significantly up-regulated in the warming group at 2 months, but down-regulated at 27 months. These two pathways are chloroalkane and chloroalkene degradation pathways and linoleic acid metabolism. By contrast, another nine metabolic pathways were significantly down-regulated in the warming group at 2 months, but upregulated at 27 months. These pathways include mRNA surveillance, N − Glycan biosynthesis, retinol metabolism, protein digestion, and absorption, biosynthesis of siderophore group nonribosomal peptides, lipopolysaccharide biosynthesis, amoebiasis, citrate cycle and peroxisome (Table S5). Most of these pathways are involved in immune-related biological functions [46–48], suggesting that gut microbiota activated the immune capacity of lizards in response to long-term warming.

LEfSe analyses indicated that the phyla Bacteroidota (*P* = 0.006), class Bacteroidia (*P* = 0.006), order Bacteroidales (*P* = 0.007), family Bacteroidaceae (*P* = 0.01), and genus *Bacteroides* (*P* = 0.01) increased significantly in the warming climate group at 27 months (Fig. 1h and Table S4). In addition, the time series analysis also confirmed that *Bacteroides* strongly responded to warming (Fig. S3e, f), and the relative abundance of *Bacteroides* was significantly higher in the warming climate group than the present climate group at 27 months (*P* = 0.01, Fig. S5e and f). These analyses indicate that *Bacteroides* play an important role in the immune response of lizards to climate warming.

### Climate warming alters the immune capacity of lizards

**Fig. 2:**
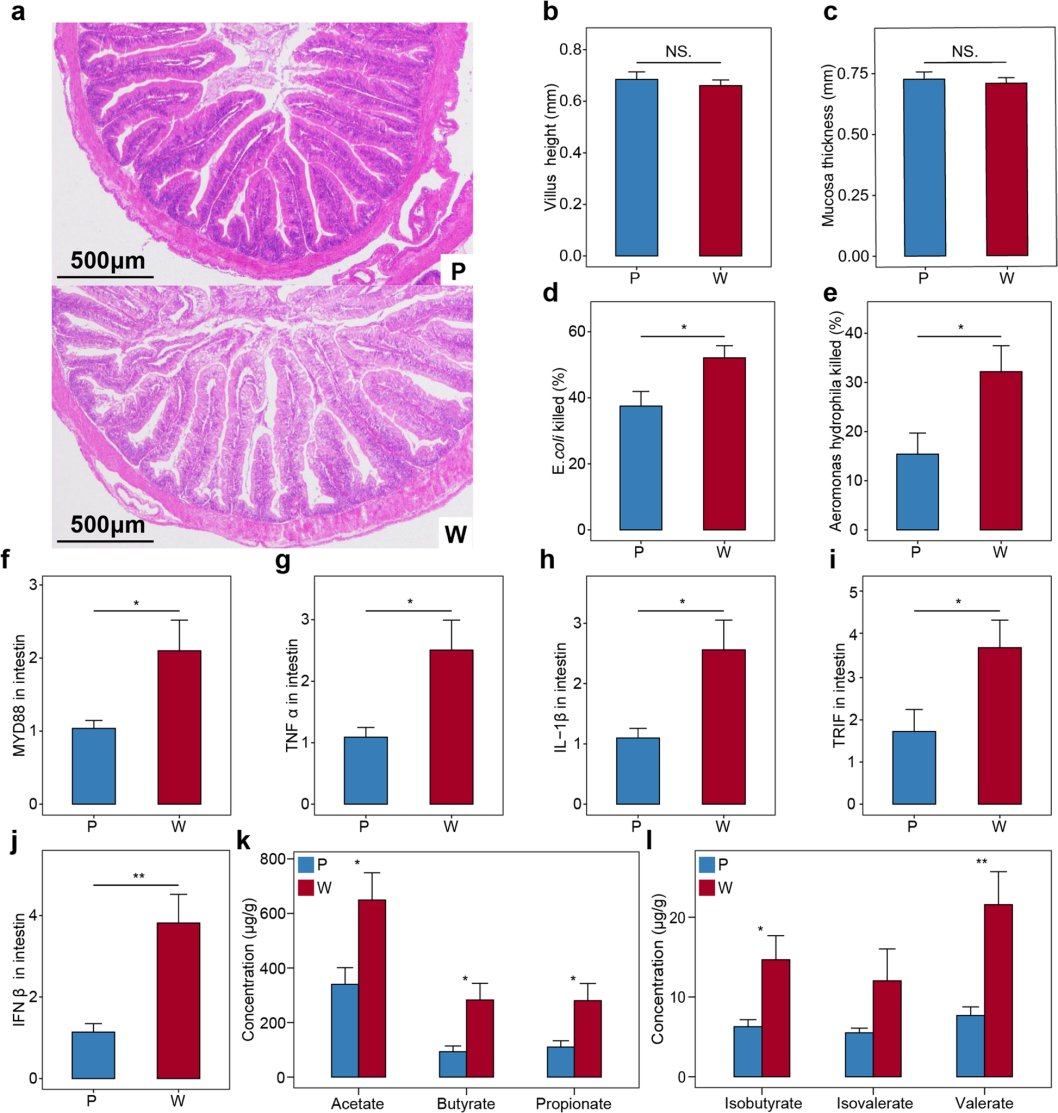
Long-term warming enhances immune capacity and concentrations of SCFAs in lizards. P represents present climate. W represents warming climate. The significance of warming effects was evaluated by a two-sided Wilcoxon rank sum test. NS non-significant; * *P* < 0.05; **P < 0.01; ***P < 0.001. **a** Tissue sections of the ileal mucosa in the 27-month group of lizards. **b**, **c** The ileal villus height and mucosal thickness in the 27-month group of lizards. **d**, **e** Antibacterial activity of serum against *Escherichia coli* and *Aeromonas hydrophila* in the 27-month group of lizards. **f**–**j** The expression of MYD88, TNFα, IL-1β, TRIF, and IFNβ in the intestines of the 27-month group of lizards. **k**, **l** The concentrations of six short-chain fatty acids (SCFAs) in lizard fecal

To elucidate the effects of climate warming on the immune capacity of lizards, we compared between-group differences in the morphology of intestinal mucosa, serum antibacterial activity, and the expression of immunity-related genes. Climate warming did not damage the intestinal barrier (Fig. 2a), with no effect on the height of the intestinal villus (*P* = 0.574, Fig. 2b) or the thickness of intestinal mucosa (*P* = 0.645, Fig. 2c). Serum antibacterial activity was significantly higher in the warming climate group than the present climate group when exposed to *Escherichia coli* (*P* = 0.031, Fig. 2d) or *Aeromonas hydrophila* (*P* = 0.036, Fig. 2e). Moreover, compared with those from the present climate group, lizards from the warming climate group showed significantly higher expression levels of immunity-related genes, including MYD88 (*P* = 0.015, Fig. 2f), TNFα (*P* = 0.015, Fig. 2g), IL-1β (*P* = 0.010, Fig. 2h), TRIF (*P* = 0.021, Fig. 2i) and IFNβ (*P* = 0.005, Fig. 2j). We then measured the concentration of six SCFAs produced by gut microbiota that affect the immune function of lizards. The total concentration of SCFAs was significantly higher in the warming climate group than the present climate group (*P* = 0.010, Table S7), as shown in acetate (*P* = 0.020), butyrate (*P* = 0.020), propionate (*P* = 0.020), isobutyrate (*P* = 0.015), and valerate (*P* = 0.001), but not in isovalerate (*P* = 0.104) (Fig. 2k, l). In total, these results show that the warming climate treatment enhanced the immune capability of lizards.

### Gut microbiota increased the immune capacity of lizards

**Fig. 3:**
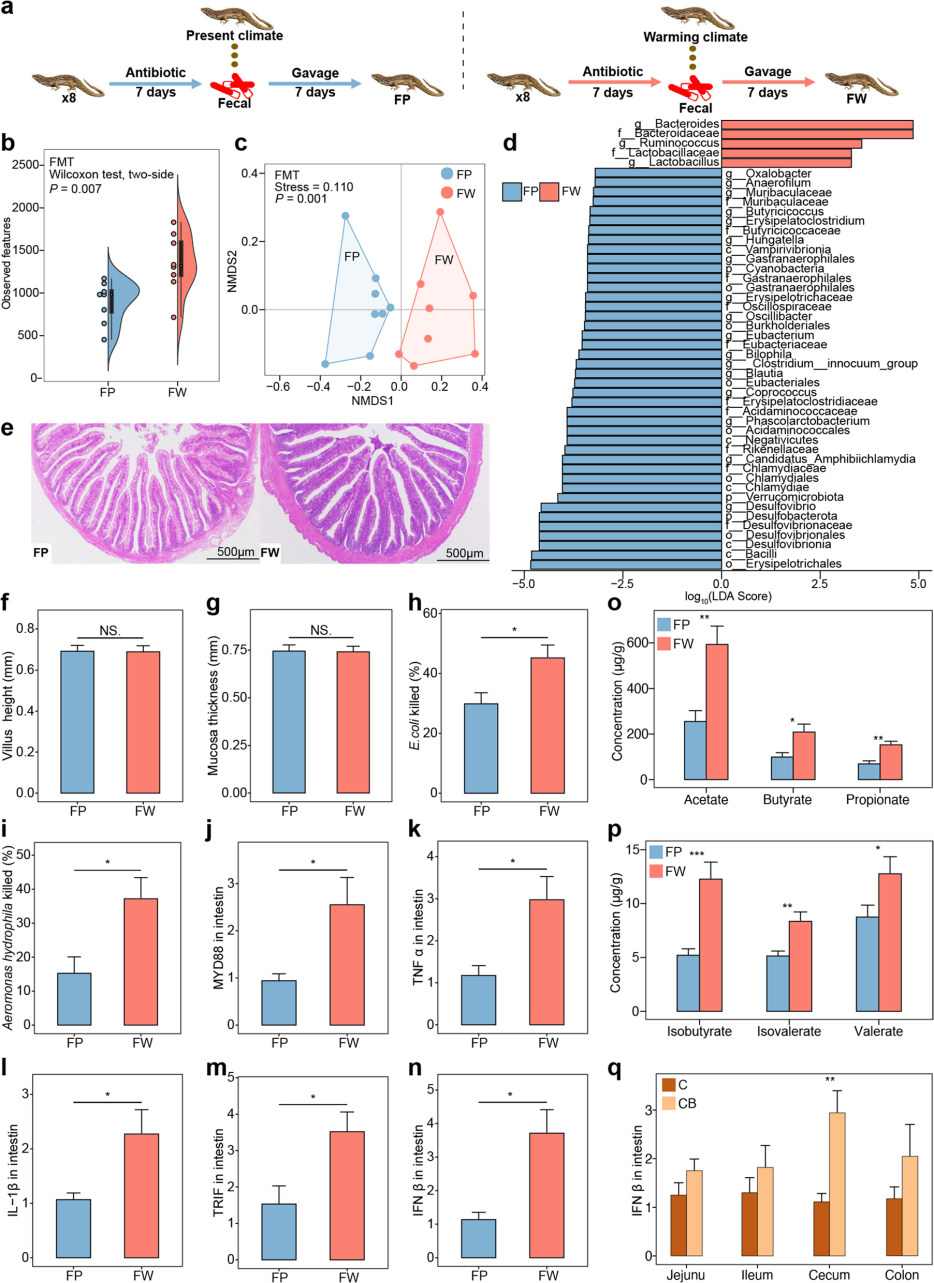
Fecal microbiota transplantation (FMT) alters immune capacity and concentrations of SCFAs in lizards. FP represents FMT of the present climate. FW represents FMT of the warming climate. C indicates “Control”, CB indicates “Colonized by *B. fragilis*”. The significance of between-treatment comparisons was evaluated by a two-sided Wilcoxon rank sum test. The significance of NMDS analysis was evaluated by PERMANOVA with 1000 permutations. NS non-significant; *P < 0.05; **P < 0.01; ***P < 0.001. **a** Design of fecal microbiota transplantation (FMT) experiments. **b** The alpha diversity index (observed features) of FMT lizards. **c** NMDS plot based on unweighted UniFrac distances of FMT lizards. **d** Biomarkers of discriminative bacteria identified by LEfSe analysis (LDA score ≥ 3.2) in the FMT lizards. **e** Tissue sections of the ileal mucosa in the FMT lizards. **f**, **g** The ileal villus height and mucosal thickness of FMT lizards. **h**, **i** Antibacterial activity of serum against *Escherichia coli* and *Aeromonas hydrophila* in FMT lizards. **j**–**n** The expression of MYD88, TNFα, IL-1β, TRIF, and IFNβ in the intestine of FMT lizards. **o**, **p** The concentrations of six short-chain fatty acids (SCFAs) in lizard fecal. **q** The expression of IFN-β in jejunum, ileum, cecum, and colon after the colonization of *B. fragilis* in lizard gut

To verify the role of gut microbiota in mediating the immune response of lizards, we transplanted fecal microbiota from present or warming climate lizards (27 months) to bacteria-restricted lizards, respectively (Fig. 3a). The alpha diversity of gut microbiota was significantly higher in lizards that received fecal microbiota from warming-climate donors than from present-climate donors (*P* = 0.007, Fig. 3b and Table S3). For beta diversity, the gut microbiota communities showed a significantly different composition between the two groups (*P* = 0.001, Fig. 3c). LEfSe methods showed that the relative abundance of the genus *Bacteroides* (*P* = 0.027) and family Bacteroidaceae (*P* = 0.027) were significantly higher in the FMT warming group compared with the FMT present group (Fig. 3d and Table S4). These results correspond with the predominant differential bacteria observed in the donor lizards.

Lizards received fecal microbiota from warming-climate donors showed no significant difference in the length of intestinal villus (*P* = 0.645, Fig. 3f) or in the thickness of intestinal mucosa (*P* = 0.563, Fig. 3g) compared to those from present-climate donors. Importantly, however, lizards that received fecal microbiota from warming-climate donors had significantly higher antibacterial activity (*P* = 0.027, Fig. 3h; *P* = 0.021, Fig. 3i) and higher expression of immune-associated genes including MYD88 (*P* = 0.038, Fig. 3j), TNFα (*P* = 0.021, Fig. 3k), IL-1β (*P* = 0.021, Fig. 3l), TRIF (*P* = 0.015, Fig. 3m), and IFNβ (*P* = 0.010, Fig. 3n), as well as higher concentrations of SCFAs (*P* = 0.004, Fig. 3o, p and Table S7), including acetate (*P* = 0.005), butyrate (*P* = 0.015), propionate (*P* = 0.003), isobutyrate (*P* = 0.0006), isovalerate (*P* = 0.001), and valerate (*P* = 0.05). These data verify that immune response activation in lizards is mediated by gut microbiota.

### *Bacteroides* alters the expression of IFN-β

To establish the specific contribution of the genus *Bacteroides* to the immune response of lizards, we analyzed the colonization of *B. fragilis* in the gut of lizards to quantify IFN-β expression levels using qRT-PCR among four segments of the intestine. The IFN-β expression in lizards colonized by *B. fragilis* was significantly increased in the cecum (*P* = 0.001, Fig. 3q), but not in other segments of the intestine. These data verify that *Bacteroides* increases IFN-β expression in the cecum of the lizard gut.

## Discussion

The interaction between host and gut microbiota is expected to shape the physiological performance and fitness of ectotherms under global climate change. Here we found that the diversity of gut microbes in a desert lizard decreased over short-term (2 months) exposure to climate warming, but increased over relatively long-term (13 and 27 months) exposure to climate warming. In turn, this enhanced the immune capacity of host lizards by activating the expression of intestinal immune-related genes. Our study thus verifies the hypothesis that alterations in the gut microbiota of lizards in a warming climate may enhance their immunity.

### Short- and long-term responses of lizard gut microbiota to climate warming

Our experiments indicate that climate warming affects the diversity, structure, and stability of the gut microbiota in reptiles. Exposure to a warming climate decreased the alpha diversity of lizard gut microbiota over 2 months, which is consistent with the findings for *Zootoca vivipara* [14], but inconsistent with the findings for *Anolis apletophallu* [49]. The pattern of reduced diversity of host microbiota under experimental warming was also found among mammals, amphibians, and birds [50, 51]. This likely reflects the negative effects of extreme temperatures on physiological functions of the host [52], and explains how species with low microbiome diversity may become more vulnerable to global warming [53–56]. Indeed, our study is the first to demonstrate that exposure to a warming climate increases the diversity of lizard gut microbiota over relatively long-term time periods (13 and 27 months). Our findings suggest that we should pay more attention to the effects of long-term climate warming on gut microbiota.

Long-term climate warming has concurrent impacts on both the environmental microbiome and the lizards’ gut microbiota. The diversity of environmental bacteria decreases under long-term warming climates, which in nature could also be induced by extreme high temperatures or drought [57, 58]. Meanwhile, the diversity of the lizard gut microbiota showed a decrease in the short term and an increase in the long term, suggesting an adaptation of the lizard’s gut microbiota to warming conditions. This adaptation could help lizards enhance their resilience to environmental changes, such as an increase in pathogenic bacteria (*Massilia* and *Cellulomonas*) in the environment [59–64]. This implies that a warming climate harbors a high risk of increased environmental pathogenic bacteria, and high gut microbial diversity may help hosts maintain physiological performance and survive harsh climatic conditions [51]. Our results suggest that increased diversity of gut microbiota is probably an adaptive response to climate change over relatively long-term periods.

### Gut microbes modulate lizard immunity in response to climate warming

Heat stress may hamper gut health, including impairment of intestinal development, gut barrier dysfunction, and improper immune responses [65, 66]. Symbiotic bacteria may help alleviate these negative effects of heat stress because they can act as a biochemical barrier to maintain the functional integrity of the intestinal barrier [67, 68]. Our study demonstrates that gut microbes may modulate the immune capacity of lizards exposed to both short- and long-term climate warming. Over short-term warming (2 months), the relative abundance of gut microbes in the genera *Desulfovibrio* and *Roseburia* increased (Fig. S5a and b), and likely played an important role in intestinal inflammatory processes [69, 70]. Additionally, the functional genes of the gut microbiota participated in the immunity regulation of lizards, including cyanoamino acid metabolism and staphylococcus aureus infection pathways that are associated with the immune system and bacterial infections [71], were significantly up-regulated in warming climate lizards (Fig. S3a). Interestingly, some pathways involved in the immune response were down-regulated over short-term warming but up-regulated over long-term warming. For example, the intestinal mucosa maintains the integrity of the intestinal barrier and health by absorbing retinoic acid produced by retinol metabolism [72]. As the primary defense mechanism, the intestinal mucosal barrier is constituted by mucins that require linkage via the glycosylation of proteoglycans, with N-glycans as a crucial component of this process [73]. In addition, lipopolysaccharide (LPS) in the intestine induces the innate immune response by binding to the Toll-like receptor 4 (TLR4)-MD-2 complex through Lipopolysaccharide Binding Protein (LBP), thereby protecting the intestinal tract from infections [48]. The upregulation of retinol metabolism, N−Glycan biosynthesis, and lipopolysaccharide biosynthesis under long-term warming can sustain a healthy intestinal barrier and induce innate immunity in lizards. These functions suggest that gut microbe modulation in response to long-term warming may play an important role in the immune response of lizards.

Our results on immunity-related gene expression demonstrate that gut microbiota may enhance the immune capacity of lizards through the following pathways: first, bacterial components such as LPS produced by gut bacteria activate the MYD88 and TRIF pathways through pathogen-associated molecular patterns [74]. Then, MYD88 initiates a signaling cascade that increases the expression of TNFα and IL-1β, which activates the lizard’s immune response [75–77]. Meanwhile, TRIF mediates the increased expression of IFN-β, which prevents excessive immune responses and enhances resistance to viral infections [21, 74]. We suggest that the gut microbiota modulation of lizard immunity increases the immune capacity of lizards as shown by the increased antibacterial activity of the serum. Additionally, due to the protection of the host intestinal barrier by gut microbes, the intestinal tissue of lizards was not damaged by long-term warming (Fig. 2a–c). Moreover, we found no decline in the survival of lizards after exposure to 27 months of warming compared to the survival of lizards in the present climate group (Fig. S6). However, the effects of climate warming have led to decreased survival of some species [78]. Thus, the higher survival rate of lizards from the climate warming treatment in our study could be attributable to enhanced immunity mediated by gut microbiota modulation, because animals with a stronger immune response are more likely to survive [79–81].

Identifying the role of specific bacteria in mediating physiological functions is critical for predicting general host health under climate warming [51, 82]. We found that the abundance of gut microbes from phyla Bacteroidota to genus *Bacteroides* increased significantly in lizards under warming-climate conditions. Previous studies have verified that certain species of *Bacteroides* are often beneficial to intestinal immunity and homeostasis, and therefore host health [83–85]. More specifically, *Bacteroides* glycolipids can activate colonic dendritic cells to secrete IFN-β through TLR4-TRIF, thereby enhancing host resistance to viral infection [21]. Because the genus *Bacteroides* contains many bacterial species, we were unable to grow every strain for testing on wild lizards. However, our experimental colonization of *B. fragilis* in the gut of lizards demonstrated that *Bacteroides* significantly enhanced the expression of IFN-β in the cecum, and therefore in turn, probably enhanced the overall immune capacity of lizards. Although we have verified the role of *Bacteroides* in enhancing host immunity, the signaling pathway by which *Bacteroides* enhances lizard immunity remains elusive and deserves further study.

SCFAs can impact the host’s immune response by influencing immune cell activity, maintaining the integrity of the intestinal barrier, and regulating inflammatory responses [86]. We also found other genera of gut microbes which may enhance the immune capacity of lizards. For example, the abundance of *Eisenbergiella* increased in the warming climate of lizards. Increased abundance of *Eisenbergiella* in the gut could be linked to an elevated production of SCFAs that are involved in anti-inflammatory gene regulation processes and promote the intestinal immune response [87, 88]. Correspondingly, the concentration of SCFAs did increase in the warming climate of lizards. Therefore, our results show that *Bacteroides* play a dominant role in modulating the immune capacity of lizards and that other bacteria may also be involved in host immune regulation.

## Conclusions

We found that the diversity of gut microbiota in a desert lizard decreased over short-term warming, but increased over long-term warming. The increased diversity of gut microbiota is likely an adaptive response to climate warming, which enhances the immune capacity of lizards facing increased risk of pathogenic bacteria. Our study mainly focused on the role of *Bacteroides* in enhancing the immune capacity of lizards; however, the role of other bacteria (e.g., *Eisenbergiella*) in modulating host physiology and behavior provides ample opportunity for future studies. Current research on how symbionts respond to climate change is limited, although increasing evidence suggests that host-microbe interactions may be critical in helping animals adapt to climate change. Our study highlights how microbial symbionts can facilitate host health and survival under climate warming and are thus important for predicting host persistence in the face of global warming.

### Supplementary Information

**Additional file 1: Fig. S1.** Photograph of chambers. **Fig. S2.** Effects of climate warming on environmental microbiota and gut microbiota of lizards. **Fig. S3.** Differential microbiota and KEGG pathways between two climate groups over 2 months and 13 months. **Fig. S4.** Differences in microbial communities at the phylum level between two climate treatment groups. **Fig. S5.** Differences in microbial communities at the genus level between two climate treatment groups. **Fig. S6.** Survival curves of lizards exposed to two climate treatments. **Table S1.** Sample information for 16S rRNA gene sequencing. **Table S2.** Statistics of 16S rRNA gene sequencing data. **Table S3.** Alpha diversity metrics. **Table S4.** The results of LEfSe. **Table S5.** The results of differential KEGG pathway analysis. **Table S6.** qRT-PCR primer sequences. **Table S7.** Quantitative analysis of short chain fatty acids. **Table S8.** Sample information for fecal microbiota transplantation (FMT) experiments. **Table S9.** Statistics of 16S rRNA gene sequencing data for fecal microbiota transplant (FMT) experiments.

## Data Availability

Raw sequence data are deposited in the Genome Sequence Archive [89] in the National Genomics Data Center [90] under accession CRA011520.
