# Supplementary material for: Gut microbiota modulation enhances the immune capacity of lizards under climate warming
Source: Microbiome. 2024 Feb 22;12:37. doi: 10.1186/s40168-023-01736-2 (PMC10882899; doi:10.1186/s40168-023-01736-2)
Supplement: Supplementary file 2 — Additional file 1: Fig. S1. Photograph of chambers. Fig. S2. Effects of climate warming on environmental microbiota and gut microbiota of lizards. Fig. S3. Differential microbiota and KEGG pathways between two climate groups over 2 months and 13 months. Fig. S4. Differences in microbial communities at the phylum level between two climate treatment groups. Fig. S5. Differences in microbial communities at the genus level between two climate treatment groups. Fig. S6. Survival curves of lizards exposed to two climate treatments. Table S1. Sample information for 16S rRNA gene sequencing. Table S2. Statistics of 16S rRNA gene sequencing data. Table S3. Alpha diversity metrics. Table S4. The results of LEfSe. Table S5. The results of differential KEGG pathway analysis. Table S6. qRT-PCR primer sequences. Table S7. Quantitative analysis of short chain fatty acids. Table S8. Sample information for fecal microbiota transplantation (FMT) experiments. Table S9. Statistics of 16S rRNA gene sequencing data for fecal microbiota transplant (FMT) experiments. [file 40168_2023_1736_MOESM1_ESM.pdf]

SUPPLEMENTARY INFORMATION

Gut microbiota modulation enhances the immune capacity of lizards under climate warming

Jing Yang[1,2], Wei-Qiang Liu[1,2], Xing-Zhi Han[1,3], Xin Hao[4], Qi-Bin Yao[1,2], Wei-Guo Du[1,*]

Corresponding author: Weiguo Du, duweiguo@ioz.ac.cn

The PDF file includes:

Supplementary results

Figs. S1 to S6

Tables S1 to S9

Supplementary Results

Alpha diversity of gut microbiota in lizards over three periods of climate warming

To illustrate the stability of our results, we calculated chao1, Faith_PD, and Shannon indexes to characterize the alpha diversity of gut microbiota in lizards (**Table S3**). We used chao1 to measure community richness and found a significant decrease after 2 months in the warming climate treatment (P = 0.010, **Fig. S2d**), but a significant increase in community richness after 13 months (P = 0.025, **Fig. S2e**) and 27 months (P = 0.001, **Fig. S2f**) in the warming climate compared to the present climate group. We used Faith_PD to measure phylogenetic diversity and found a significant decrease after 2 months (P = 0.016, **Fig. S2g**), a significant increase after 27 months (P = 0.001, **Fig. S2i**), but no significant difference after 13 months (P = 0.201, **Fig. S2h**) in the warming climate group compared to the present climate group. The Shannon index was used to measure community diversity and we found a significant increase after 27 months (P = 0.002, **Fig. S2l**), but no significant difference after 2 months (P = 0.748, **Fig. S2j**) or 13 months (P = 0.261, **Fig. S2k**) in the warming climate group compared to the present climate group. Due to the unequal number of females and males in the climate treatments, we examined the effects of gender on the gut microbiota of lizards from the same climate. NMDS analysis showed that there was no significant difference in the composition of gut microbiota between genders in the present (P = 0.398, stress = 0.110) or warming climate (P = 0.332, stress = 0.110) treatments.

Biomarker and functional prediction of gut microbiota in lizards

In order to determine which functional pathways of lizard gut bacterial communities were involved in warming climate adaptation, we performed KEGG metabolic pathway prediction analysis. Compared to lizards in the 2-month present climate treatment, the warming climate lizards significantly up-regulated seven metabolic pathways, including cyanoamino acid metabolism, staphylococcus aureus infection, and chlorocyclohexane and chlorobenzene degradation pathways (**Fig. S3a** and **Table S5**). Lizards from the 13-month warming climate group significantly up-regulated five metabolic pathways, including endocytosis, ECM−receptor interaction, and nitrotoluene degradation (**Fig. S3c** and **Table S5**).

To screen for biomarkers in lizard gut microbiota, we conducted LEfSe analyses and time series analysis (**Table S4**). LDA score > 3.2 showed that warming influenced lizard gut microbiota, from phylum to genus. Taxa from the phylum Firmicutes, class Clostridia, genus *Roseburia*, and genus

Massilia were more frequently observed in the 2-month warming climate group (**Fig. S3b)**. Taxa from genus *Romboutsia*, family S085, genus S085, and order S085 were more frequently observed in the 13-month warming climate group (**Fig. S3d**). In the time series analysis, we found that changes in relative abundances of family Erysipelotrichaceae, genus *Akkermansia*, class Clostridia, order Lachnospirales, order Verrucomicrobiales and phylum Firmicutes in warming climate lizards were not correlated with abundances in the present climate lizards, and that differences in abundance between the two climate groups were large (**Fig. S3e**). The abundance of order Burkholderiales and family Enterobacteriaceae fluctuated over short- to long-term exposure to warming, decreasing at 2 months but increasing at 13 months, then decreasing at 27 months. The abundance of Class Bacteroidia and genus *Bacteroides* decreased slightly at 2 months and then gradually increased until 27 months (**Fig. S3f**).

Changes in the relative abundance of gut microbiota in lizards during three warming periods

The bacterial communities in lizard gut microbiota belonged to 41 different phyla, with Firmicutes and Bacteroidota dominating the bacterial community in gut microbiota. The relative abundance of phylum Firmicutes (P = 0.003) significantly increased in lizards from the 2-month warming climate group compared to lizards from the present climate group; in contrast, the relative abundance of Desulfobacterota (P = 0.019) and Proteobacteria (P = 0.003) showed a significant decrease (**Fig. S4a** and **b**). The relative abundance of Bacteroidetes (P = 0.012, **Fig. S4e** and **f**) was significantly higher in the 27-month warming climate group than the present climate group, but the two groups did not differ in Bacteroidetes abundance after 13 months (**Fig. S4c** and **d**).

At the generic level, the relative abundance of *Desulfovibrio* (P = 0.012) and *Odoribacter* (P = 0.040) were significantly decreased in the 2-month warming climate, but the abundance of *Roseburia* (P = 0.026) was significantly increased (**Fig. S5a** and **b**). The relative abundances of *Bacteroides* (P = 0.010), *Eisenbergiella* (P = 0.012) and *Parabacteroides* (P = 0.001) were significantly higher in the 27-month warming climate group than the present climate group of lizards (**Fig. S5e** and **f**). The relative abundance of *Clostridium innocuum* was significantly decreased in the 13-month warming climate lizards (P = 0.031, **Fig. S5c** and **d**).

Supplementary figures

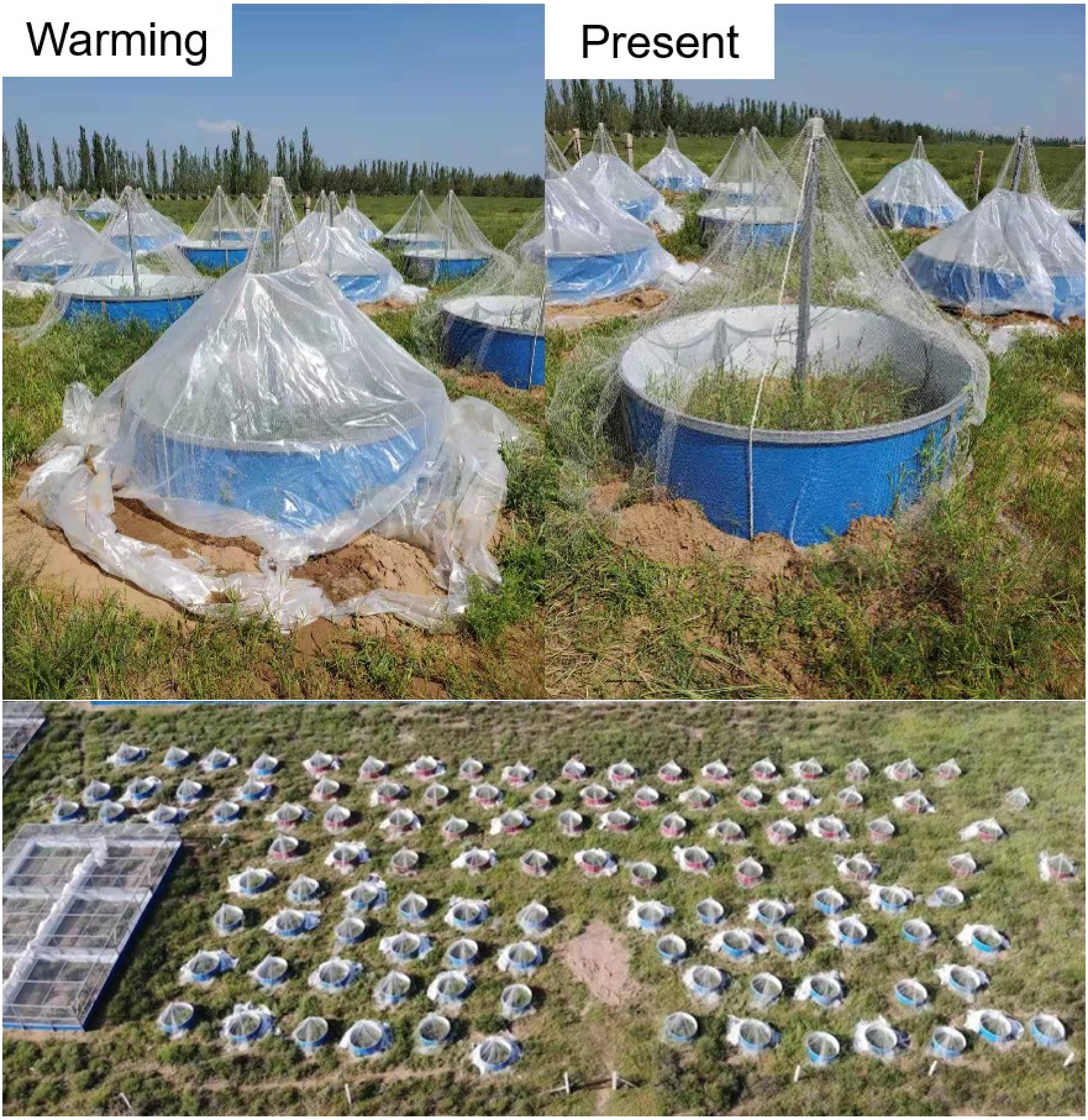

Fig. S1. Photograph of chambers. Upper shows two types of chambers (left is warming; right is present), and lower shows a top view of all chambers.

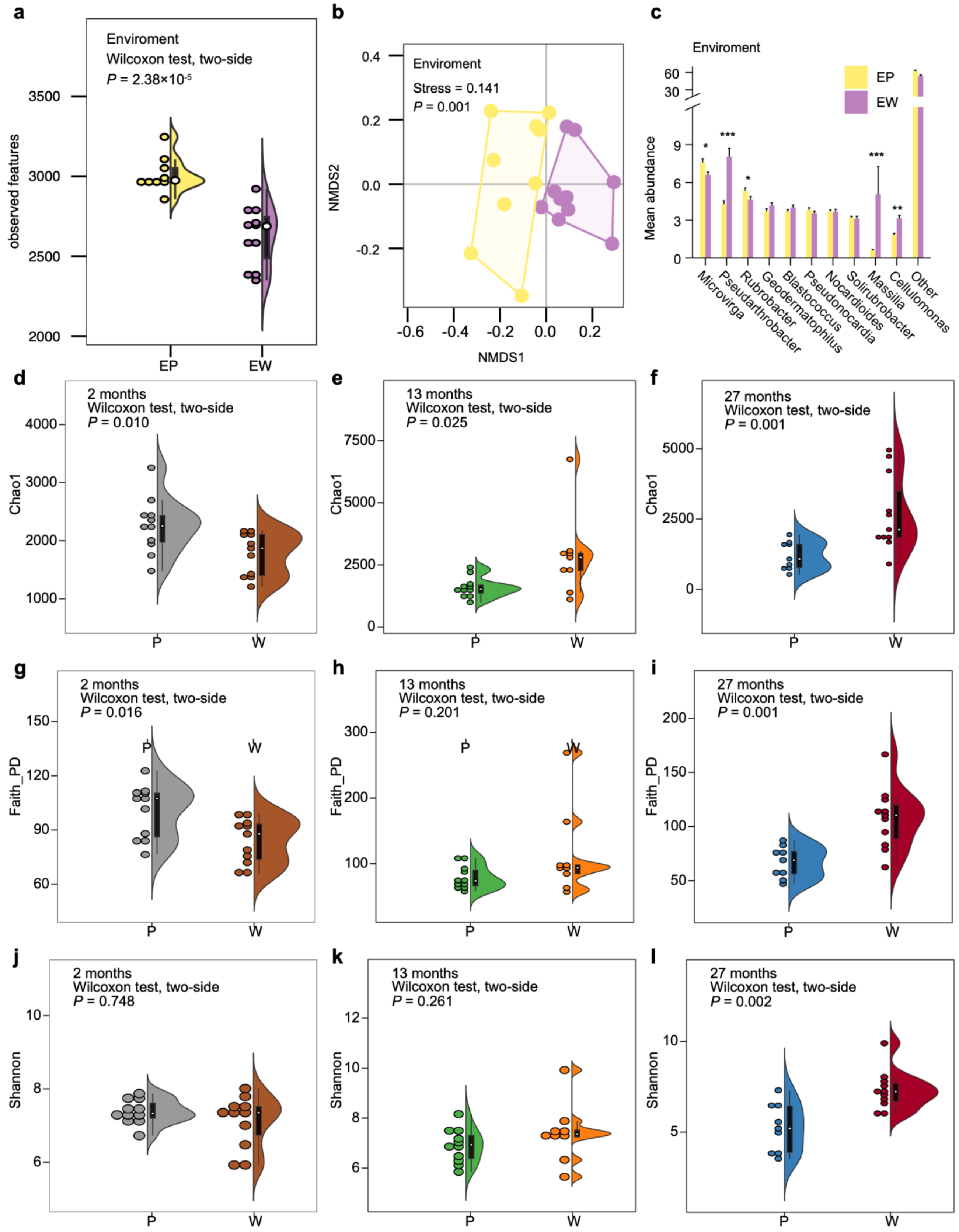

Fig. S2. Effects of climate warming on environmental microbiota and gut microbiota of lizards. EP represent environment of present climate. EW represent environment of warming climate. P represents present climate. W represents warming climate. The significance of warming effects was evaluated by a two-sided

Wilcoxon rank sum test. * *P* < 0.05; ** *P* < 0.01; *** *P* < 0.001. The significance of non-metric multidimensional scaling (NMDS) analysis was evaluated by PERMANOVA with 1000 permutations.

(**a)** The alpha diversity index (observed features) of environmental microbiota.

(**b**) NMDS plot based on unweighted UniFrac distances of environmental microbiota.

(**c**) Bar plot showing the relative abundance of the top 10 genera of environmental microbiota in present and warming climate treatments.

(**d-f**) The alpha diversity index (Chao1) of lizard gut microbiota at 2 months (**d**), 13 months (**e**), and 27 months (**f**).

(**g-i**) The alpha diversity index (Faith_PD) of lizard gut microbiota at 2 months (**g**), 13 months (**h**), and 27 months (**i**).

(**j-l**) The alpha diversity index (shannon) of lizard gut microbiota at 2 months (**j**), 13 months (**k**), and 27 months (**l**).

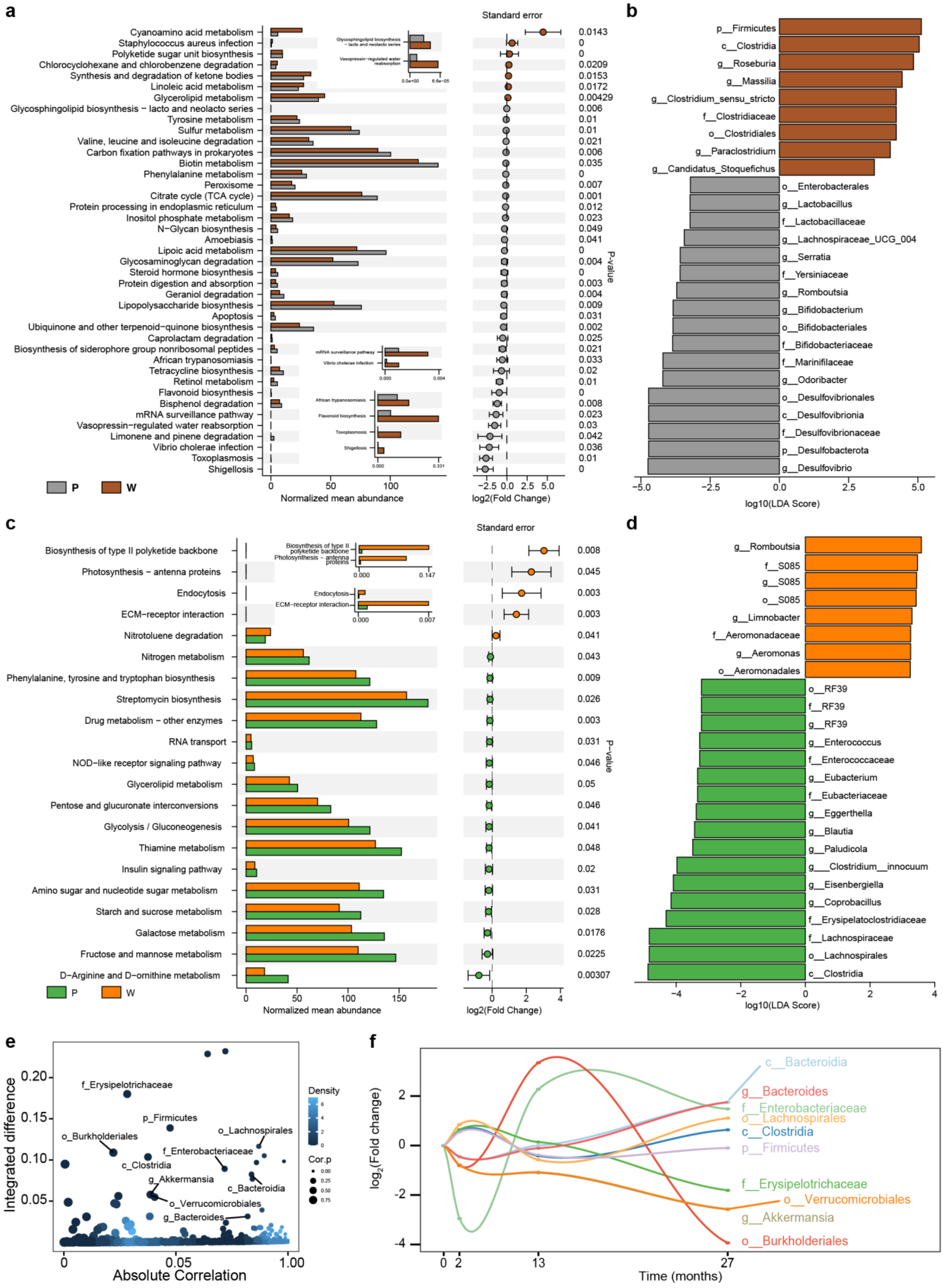
a
Cyanoamino acid metabolism
Staphylococcus aureus infection
Polyketide sugar unit biosynthesis
Chlorocyclohexane and chlorobenzene degradation
Synthesis and degradation of ketone bodies
Linoleic acid metabolism
Glycerolipid metabolism
Glycosphingolipid biosynthesis − lacto and neolacto series
Tyrosine metabolism
Sulfur metabolism
Valine, leucine and isoleucine degradation
Carbon fixation pathways in prokaryotes
Biotin metabolism
Phenylalanine metabolism
Peroxisome
Citrate cycle (TCA cycle)
Protein processing in endoplasmic reticulum
Inositol phosphate metabolism
N−Glycan biosynthesis
Amoebiasis
Lipoic acid metabolism
Glycosaminoglycan degradation
Steroid hormone biosynthesis
Protein digestion and absorption
Geraniol degradation
Lipopolysaccharide biosynthesis
Apoptosis
Ubiquinone and other terpenoid−quinone biosynthesis
Caprolactam degradation
Biosynthesis of siderophore group nonribosomal peptides
African trypanosomiasis
Tetracycline biosynthesis
Retinol metabolism
Flavonoid biosynthesis
Bisphenol degradation
mRNA surveillance pathway
Vasopressin−regulated water reabsorption
Limonene and pinene degradation
Vibrio cholerae infection
Toxoplasmosis
Shigellosis
P
W
Normalized mean abundance
Standard error
log2(Fold Change)
P-value
0.0143
0
0
0.0209
0.0153
0.0172
0.00429
0.006
0.01
0.01
0.021
0.006
0.035
0
0.007
0.001
0.012
0.023
0.049
0.041
0
0.004
0
0.003
0.004
0.009
0.031
0.002
0.025
0.021
0.033
0.02
0.01
0
0.008
0.023
0.03
0.042
0.036
0.01
0
b
p__Firmicutes
c__Clostridia
g__Roseburia
g__Massilia
g__Clostridium_sensu_stricto
f__Clostridiaceae
o__Clostridiales
g__Paraclostridium
g__Candidatus_Stoquefichus
o__Enterobacterales
g__Lactobacillus
f__Lactobacillaceae
g__Lachnospiraceae_UCG_004
g__Serratia
f__Yersiniaceae
g__Romboutsia
g__Bifidobacterium
o__Bifidobacteriales
f__Bifidobacteriaceae
f__Marinifilaceae
g__Odoribacter
o__Desulfovibrionales
c__Desulfovibrionia
f__Desulfovibrionaceae
p__Desulfobacterota
g__Desulfovibrio
log10(LDA Score)
c
Biosynthesis of type II polyketide backbone
Photosynthesis − antenna proteins
Endocytosis
ECM−receptor interaction
Nitrotoluene degradation
Nitrogen metabolism
Phenylalanine, tyrosine and tryptophan biosynthesis
Streptomycin biosynthesis
Drug metabolism − other enzymes
RNA transport
NOD−like receptor signaling pathway
Glycerolipid metabolism
Pentose and glucuronate interconversions
Glycolysis / Gluconeogenesis
Thiamine metabolism
Insulin signaling pathway
Amino sugar and nucleotide sugar metabolism
Starch and sucrose metabolism
Galactose metabolism
Fructose and mannose metabolism
D−Arginine and D−ornithine metabolism
P
W
Normalized mean abundance
Standard error
log2(Fold Change)
P-value
0.008
0.045
0.003
0.003
0.041
0.043
0.009
0.026
0.003
0.031
0.046
0.05
0.046
0.041
0.048
0.02
0.031
0.028
0.0176
0.0225
0.00307
d
g__Romboutsia
f__S085
g__S085
o__S085
g__Limnobacter
f__Aeromonadaceae
g__Aeromonas
o__Aeromonadales
o__RF39
f__RF39
g__RF39
g__Enterococcus
f__Enterococcaceae
g__Eubacterium
f__Eubacteriaceae
g__Eggerthella
g__Blautia
g__Paludicola
g__Clostridium__innocuum
g__Eisenbergiella
g__Coprobacillus
f__Erysipelatoclostridiaceae
f__Lachnospiraceae
o__Lachnospirales
c__Clostridia
log10(LDA Score)
e
Integrated difference
Absolute Correlation
f_Erysipelotrichaceae
p_Firmicutes
o_Burkholderiales
o_Lachnospirales
f_Enterobacteriaceae
c_Clostridia
g_Akkermansia
c_Bacteroidia
o_Verrucomicrobiales
g_Bacteroides
Density
Cor.p
f
log2(Fold change)
Time (months)
c__Bacteroidia
g__Bacteroides
f__Enterobacteriaceae
o__Lachnospirales
c__Clostridia
p__Firmicutes
f__Erysipelotrichaceae
o__Verrucomicrobiales
g__Akkermansia
o__Burkholderiales

Fig. S3. Differential microbiota and KEGG pathways between two climate groups over 2 months and 13 months. P represents present climate. W represents warming climate.

(**a**, **c**) The functional profiles of gut microbiota identified by PICRUSt2 using KEGG database in different climates at 2 months (**a**) and 13 months (**c**).

(**b**, **d**) Biomarkers of discriminative bacteria in different climates identified by LEfSe analysis (LDA score ≥3.2) at 2 months (**b**) and 13 months (**d**).

(**e**) The score is plotted as A (1 - [absolute correlation coefficient]) versus B (integrated difference between the curves). Important taxonomic groups are marked with the taxa name in black. The color indicates the density of data points.

(**f**) Temporal variation of specific bacteria communities. Y-axis represents the $\log_2$ (Fold change) of the warming climate group relative to the present climate group. X-axis represents the sampling time. The curve is simulated by loess with span as 0.8.

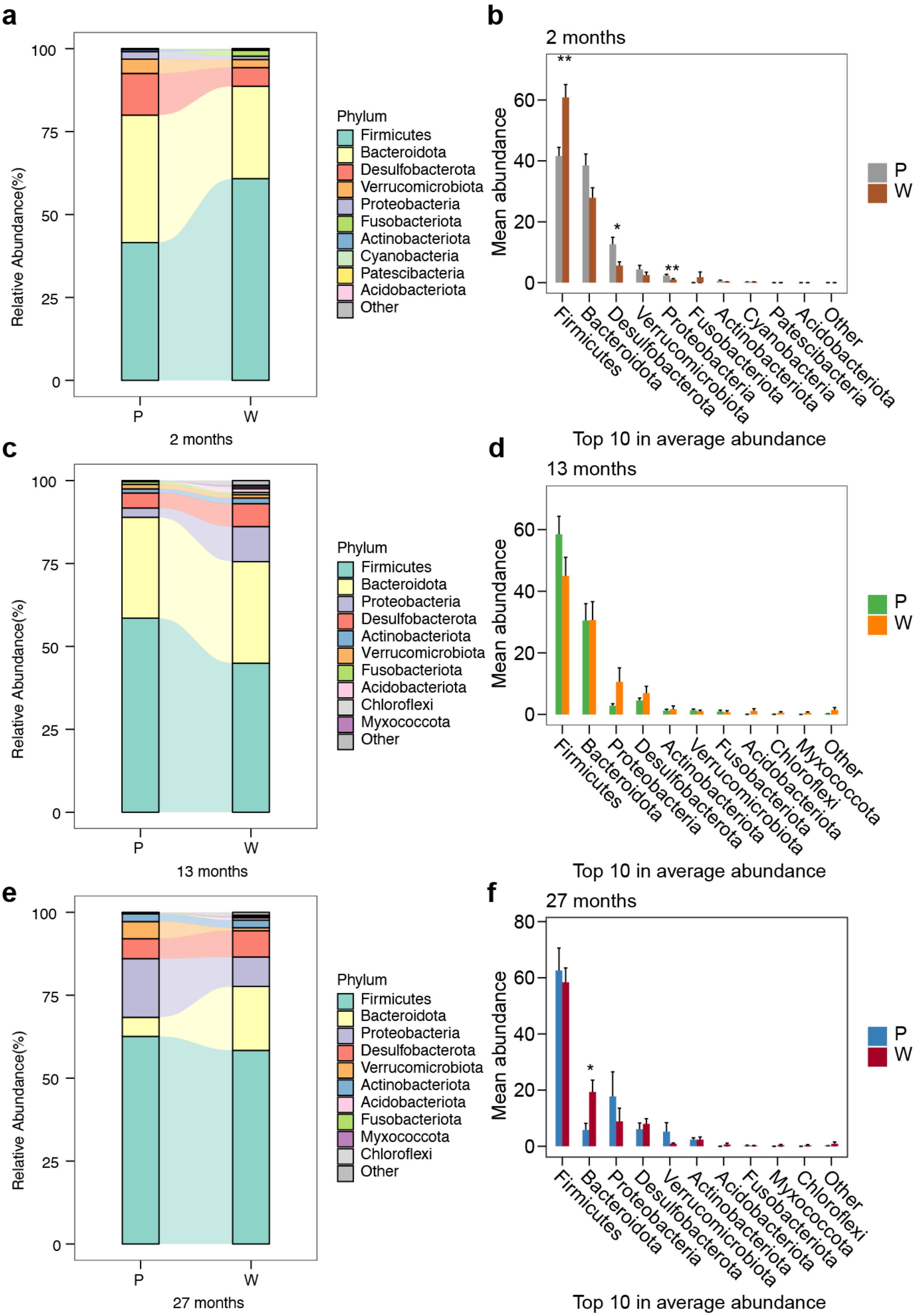
a
Relative Abundance(%)
100
75
50
25
0
P
W
2 months
Phylum
Firmicutes
Bacteroidota
Desulfobacterota
Verrucomicrobiota
Proteobacteria
Fusobacteriota
Actinobacteriota
Cyanobacteria
Patescibacteria
Acidobacteriota
Other
b
2 months
Mean abundance
60
40
20
0
**
*
**
Firmicutes
Bacteroidota
Desulfobacterota
Verrucomicrobiota
Proteobacteria
Fusobacteriota
Actinobacteriota
Cyanobacteria
Patescibacteria
Acidobacteriota
Other
P
W
Top 10 in average abundance
c
Relative Abundance(%)
100
75
50
25
0
P
W
13 months
Phylum
Firmicutes
Bacteroidota
Proteobacteria
Desulfobacterota
Actinobacteriota
Verrucomicrobiota
Fusobacteriota
Acidobacteriota
Chloroflexi
Myxococcota
Other
d
13 months
Mean abundance
60
40
20
0
Firmicutes
Bacteroidota
Proteobacteria
Desulfobacterota
Actinobacteriota
Verrucomicrobiota
Fusobacteriota
Acidobacteriota
Chloroflexi
Myxococcota
Other
P
W
Top 10 in average abundance
e
Relative Abundance(%)
100
75
50
25
0
P
W
27 months
Phylum
Firmicutes
Bacteroidota
Proteobacteria
Desulfobacterota
Verrucomicrobiota
Actinobacteriota
Acidobacteriota
Fusobacteriota
Myxococcota
Chloroflexi
Other
f
27 months
Mean abundance
80
60
40
20
0
*
Firmicutes
Bacteroidota
Proteobacteria
Desulfobacterota
Verrucomicrobiota
Actinobacteriota
Acidobacteriota
Fusobacteriota
Myxococcota
Chloroflexi
Other
P
W
Top 10 in average abundance

Fig. S4. Differences in microbial communities at the phylum level between two climate treatment groups. P represents present climate. W represents warming climate. The significance of warming effects was evaluated by a two-sided Wilcoxon rank sum test. * $P < 0.05$; ** $P < 0.01$; *** $P < 0.001$.

(**a**, **c**, **e**) Alluvium plot of the relative abundance of the top 10 phyla of lizard gut microbiota at 2 months (**a**), 13 months (**c**) and 27 months (**e**).

(**b**, **d**, **f**) Bar plot of the relative abundance in the top 10 phyla of lizard gut microbiota at 2 months (**b**), 13 months (**d**) and 27 months (**f**).

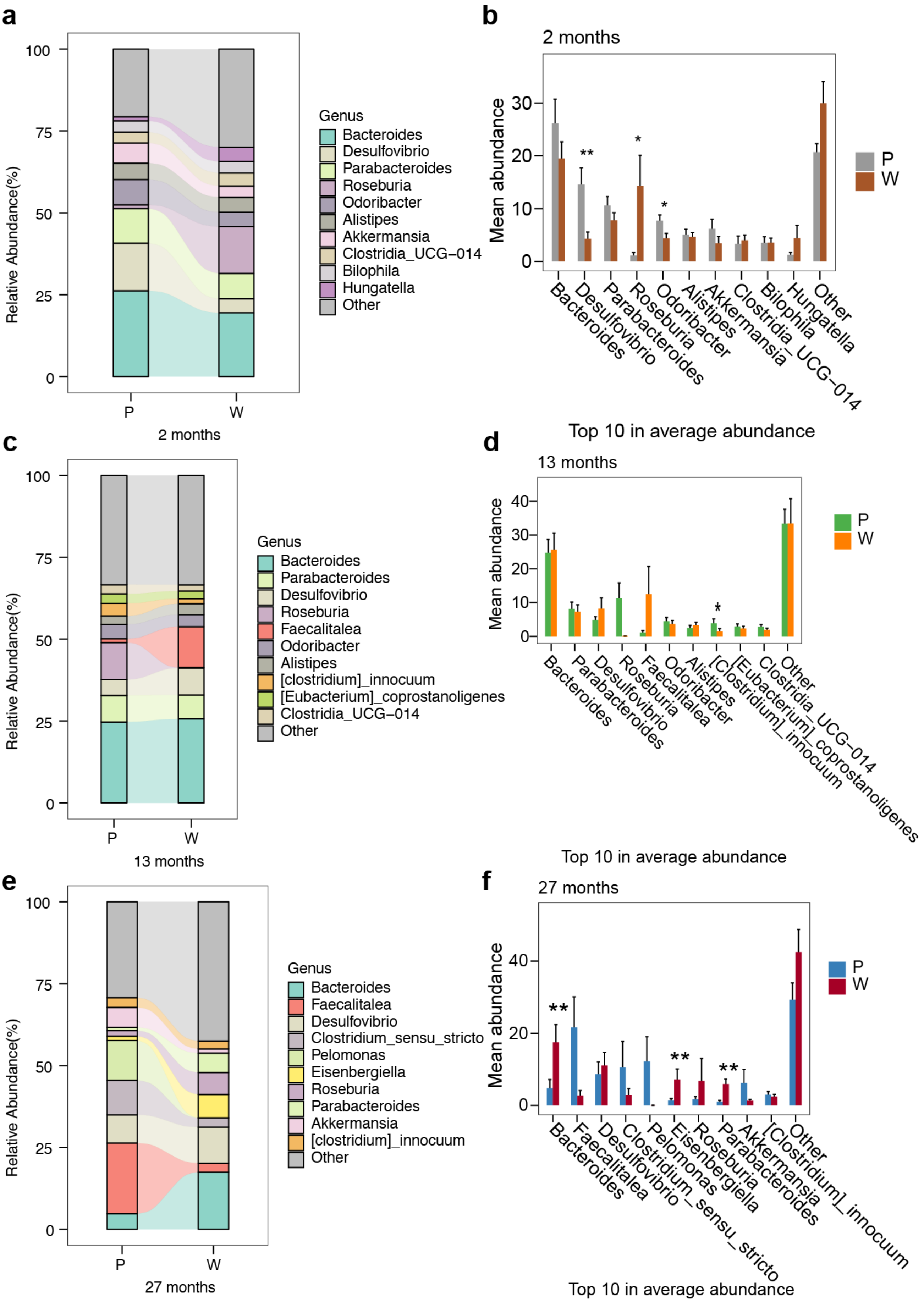
a
100
75
50
25
0
Relative Abundance(%)
P
W
2 months
Genus
Bacteroides
Desulfovibrio
Parabacteroides
Roseburia
Odoribacter
Alistipes
Akkermansia
Clostridia_UCG–014
Bilophila
Hungatella
Other
b
2 months
Mean abundance
30
20
10
0
**
*
*
P
W
Bacteroides
Desulfovibrio
Parabacteroides
Roseburia
Odoribacter
Alistipes
Akkermansia
Clostridia_UCG–014
Bilophila
Hungatella
Other
Top 10 in average abundance
c
Relative Abundance(%)
P
W
13 months
Genus
Bacteroides
Parabacteroides
Desulfovibrio
Roseburia
Faecalitalea
Odoribacter
Alistipes
[clostridium]_innocuum
[Eubacterium]_coprostanoligenes
Clostridia_UCG–014
Other
d
13 months
Mean abundance
40
30
20
10
0
*
P
W
Bacteroides
Parabacteroides
Desulfovibrio
Roseburia
Faecalitalea
Odoribacter
Alistipes
[Clostridium]_innocuum
[Eubacterium]_coprostanoligenes
Clostridia_UCG–014
Other
Top 10 in average abundance
e
Relative Abundance(%)
P
W
27 months
Genus
Bacteroides
Faecalitalea
Desulfovibrio
Clostridium_sensu_stricto
Pelomonas
Eisenbergiella
Roseburia
Parabacteroides
Akkermansia
[clostridium]_innocuum
Other
f
27 months
Mean abundance
40
20
0
**
**
**
P
W
Bacteroides
Faecalitalea
Desulfovibrio
Clostridium_sensu_stricto
Pelomonas
Eisenbergiella
Roseburia
Parabacteroides
Akkermansia
[Clostridium]_innocuum
Other
Top 10 in average abundance

Fig. S5. Differences in microbial communities at the genus level between two climate treatment groups. P represents present climate. W represents warming climate. The significance of warming effects was evaluated by a two-sided Wilcoxon rank sum test. * $P < 0.05$; ** $P < 0.01$; *** $P < 0.001$.

(**a**, **c**, **e**) Alluvium plot showing the relative abundance of the top 10 genera of lizard gut microbiota at 2 months (**a**), 13 months (**c**) and 27 months (**e**).

(**b**, **d**, **f**) Bar plot showing the relative abundance of the top 10 genera of lizard gut microbiota at 2 months (**b**), 13 months (**d**) and 27 months (**f**).

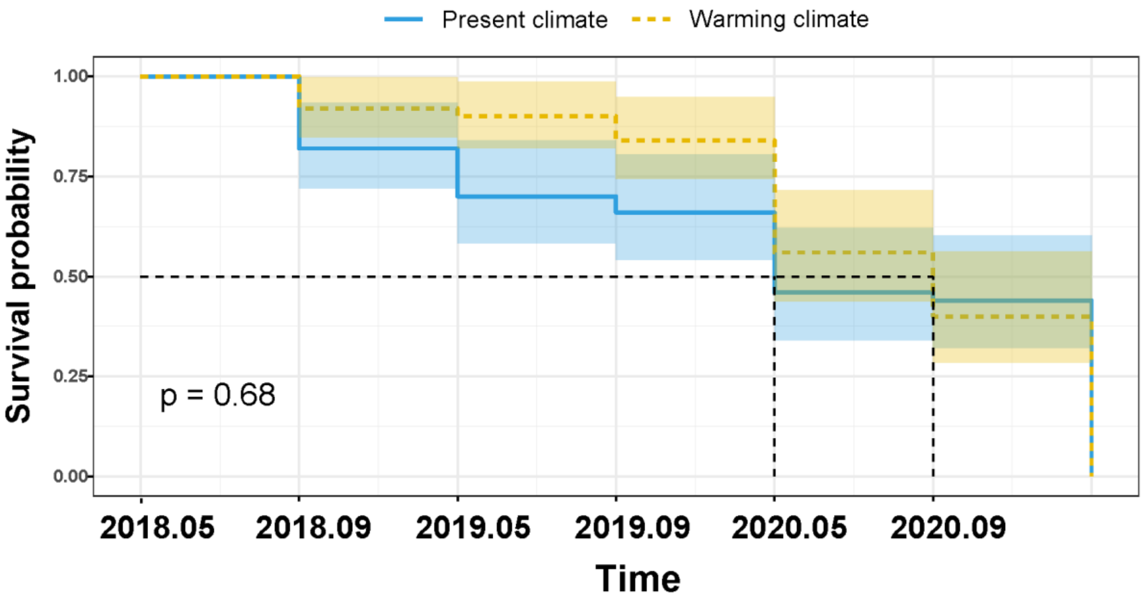

Fig. S6. Survival curves of lizards exposed to two climate treatments. The solid blue line represents the median survival of lizards in the present climate group, and the yellow dashed line represents the median survival of lizards in the warming climate group. Shadows represent 95% confidence intervals. The significance of warming effects was evaluated by generalized linear mixed models with binomial distributions.

Supplementary tables

Table S1. Sample information for 16S rRNA gene sequencing

NO.	Name	Enclosure	Climate	Sex	Treat Time	Treat Group	Snout-vent Length	Body Mass
1	2m-P1	RC2	present	M	2021.06-2021.08	2 months	57.99	5.607
2	2m-P2	RC2	present	M	2021.06-2021.08	2 months	70.5	10.025
3	2m-P3	RC12	present	M	2021.06-2021.08	2 months	62.27	6.333
4	2m-P4	RC12	present	M	2021.06-2021.08	2 months	66.18	8.488
5	2m-P5	RC14	present	F	2021.06-2021.08	2 months	69.66	8.748
6	2m-P6	RC14	present	M	2021.06-2021.08	2 months	62.18	6.812
7	2m-P7	RC14	present	M	2021.06-2021.08	2 months	63.92	6.754
8	2m-P8	RC16	present	F	2021.06-2021.08	2 months	61.54	6.792
9	2m-P9	RC16	present	M	2021.06-2021.08	2 months	62.77	7.269
10	2m-P10	RC18	present	F	2021.06-2021.08	2 months	61.68	6.464
11	2m-P11	RC18	present	M	2021.06-2021.08	2 months	68.58	8.796
12	2m-W1	RH2	warming	M	2021.06-2021.08	2 months	62.72	8.426
13	2m-W2	RH4	warming	F	2021.06-2021.08	2 months	66.23	8.534
14	2m-W3	RH4	warming	M	2021.06-2021.08	2 months	68.22	9.538
15	2m-W4	RH6	warming	F	2021.06-2021.08	2 months	68.04	9.689
16	2m-W5	RH6	warming	M	2021.06-2021.08	2 months	62.27	7.203
17	2m-W6	RH8	warming	F	2021.06-2021.08	2 months	67.56	8.292
18	2m-W7	RH10	warming	F	2021.06-2021.08	2 months	64.06	7.718
19	2m-W8	RH14	warming	F	2021.06-2021.08	2 months	67.42	6.869
20	2m-W9	RH14	warming	M	2021.06-2021.08	2 months	66.28	8.783
21	2m-W10	RH18	warming	M	2021.06-2021.08	2 months	61.64	5.714
22	2m-W11	RH18	warming	M	2021.06-2021.08	2 months	65.46	6.657
23	1y-P1	BC6	present	F	2019.07-2020.08	13 months	70.45	7.314
24	1y-P2	BC2	present	M	2019.07-2020.08	13 months	76.39	8.436
25	1y-P3	BC4	present	F	2019.07-2020.08	13 months	76.73	8.235
26	1y-P4	BC8	present	M	2019.07-2020.08	13 months	72.98	7.722
27	1y-P5	BC4	present	F	2019.07-2020.08	13 months	78.22	10.34
28	1y-P6	BC2	present	F	2019.07-2020.08	13 months	71.65	7.606
29	1y-P7	BC8	present	F	2019.07-2020.08	13 months	73.98	6.515
30	1y-P8	BC6	present	F	2019.07-2020.08	13 months	75.59	7.465
31	1y-P9	BC18	present	F	2019.07-2020.08	13 months	80.17	8.666
32	1y-P10	BC2	present	M	2019.07-2020.08	13 months	73.28	8.423
33	1y-P11	BC8	present	M	2019.07-2020.08	13 months	80.07	11.952
34	1y-W1	BH2	warming	F	2019.07-2020.08	13 months	71.26	7.891
35	1y-W2	BH18	warming	F	2019.07-2020.08	13 months	78.6	8.19
36	1y-W3	BH4	warming	M	2019.07-2020.08	13 months	76.24	8.405
37	1y-W4	BH6	warming	M	2019.07-2020.08	13 months	75.1	8.755
38	1y-W5	BH2	warming	M	2019.07-2020.08	13 months	71.26	7.891
39	1y-W6	BH12	warming	F	2019.07-2020.08	13 months	73.94	8.482
40	1y-W7	BH8	warming	F	2019.07-2020.08	13 months	76.29	8.199
41	1y-W8	BH10	warming	F	2019.07-2020.08	13 months	79.99	10.938
42	1y-W9	BH10	warming	F	2019.07-2020.08	13 months	75.29	8.708

NO.	Name	Enclosure	Climate	Sex	Treat Time	Treat Group	Snout-vent Length	Body Mass
43	3y-P1	RC2	present	M	2018.05-2020.08	27 months	75.39	9.228
44	3y-P2	RC12	present	M	2018.05-2020.08	27 months	73.41	8.649
45	3y-P3	RC20	present	M	2018.05-2020.08	27 months	76.48	9.038
46	3y-P4	RC2	present	F	2018.05-2020.08	27 months	70.32	8.085
47	3y-P5	RC4	present	F	2018.05-2020.08	27 months	75.39	9.314
48	3y-P6	RC8	present	F	2018.05-2020.08	27 months	73.92	7.395
49	3y-P7	RC8	present	F	2018.05-2020.08	27 months	75.85	7.901
50	3y-P8	RC10	present	M	2018.05-2020.08	27 months	72.93	7.986
51	3y-P9	RC10	present	F	2018.05-2020.08	27 months	71.39	5.898
52	3y-W1	RH16	warming	F	2018.05-2020.08	27 months	79.78	9.01
53	3y-W2	RH10	warming	F	2018.05-2020.08	27 months	75.12	7.588
54	3y-W3	RH14	warming	F	2018.05-2020.08	27 months	71.43	6.988
55	3y-W4	RH6	warming	F	2018.05-2020.08	27 months	74.1	7.959
56	3y-W5	RH4	warming	F	2018.05-2020.08	27 months	76.66	10.037
57	3y-W6	RH10	warming	M	2018.05-2020.08	27 months	71.52	7.359
58	3y-W7	RH10	warming	M	2018.05-2020.08	27 months	78.48	9.266
59	3y-W8	RH14	warming	F	2018.05-2020.08	27 months	71.3	5.39
60	3y-W9	RH16	warming	M	2018.05-2020.08	27 months	74.64	6.663
61	3y-W10	RH16	warming	M	2018.05-2020.08	27 months	76.08	9.614
62	3y-W11	RH18	warming	F	2018.05-2020.08	27 months	71.09	6.204
63	n1	/	/	M	/	/	66.37	5.669
64	n2	/	/	M	/	/	60.88	5.286
65	n3	/	/	M	/	/	64.22	5.509
66	n4	/	/	M	/	/	62.38	5.926
67	n5	/	/	F	/	/	67.93	7.296
68	n6	/	/	F	/	/	64.12	6.448
69	n7	/	/	F	/	/	61.5	5.069
70	n8	/	/	F	/	/	63.98	6.769
71	n9	/	/	M	/	/	69.21	8.424
72	n10	/	/	F	/	/	66.72	6.209

Table S2. Statistics of 16S rRNA gene sequencing data

Sample	Input	Filtered	Denoised	Merged	Non-chimeric
2m-P1	92499	85971	83392	69419	48500
2m-P2	111701	105230	102738	90902	66868
2m-P3	123618	115958	113701	103360	73765
2m-P4	130808	122479	119602	103123	66216
2m-P5	109365	102566	100218	85930	64133
2m-P6	120323	112983	109917	92558	57870
2m-P7	131110	122190	118571	97444	68687
2m-P8	126175	117741	115675	104943	69861
2m-P9	116990	109847	107159	91719	57305
2m-P10	102837	95747	92869	75515	53021
2m-P11	117338	109975	108265	98723	83749

Sample	Input	Filtered	Denoised	Merged	Non-chimeric
2m-W1	106826	100435	98065	83290	64922
2m-W2	106837	100023	98188	86336	63751
2m-W3	140564	131919	129089	110978	87653
2m-W4	108561	101640	98727	82148	57941
2m-W5	147873	138081	135670	117927	89807
2m-W6	111446	103909	101579	87634	65798
2m-W7	147367	137824	135648	122788	98532
2m-W8	146273	137634	134784	118874	86390
2m-W9	119140	111149	109851	100958	86141
2m-W10	105430	98489	96893	88266	65380
2m-W11	111963	104081	102378	93083	67647
1y-P1	96899	85386	83663	74848	56824
1y-P2	65844	57429	55357	45764	36847
1y-P3	119559	103063	101066	89606	73770
1y-P4	139415	122261	119920	103071	77618
1y-P5	101043	88367	86811	77535	58637
1y-P6	133515	117124	114714	97628	73573
1y-P7	127044	109541	107006	95022	72522
1y-P8	117826	102622	100777	91149	64007
1y-P9	134252	119855	117849	107120	80582
1y-P10	100265	87277	85787	78297	70059
1y-P11	101175	88522	86985	80121	74461
1y-W1	135537	117254	115245	103827	83167
1y-W2	157851	138831	137002	125881	92568
1y-W3	111960	99678	95306	78551	71776
1y-W4	104889	93428	91195	79006	56392
1y-W5	140125	120215	118266	105707	101195
1y-W6	174801	150477	147788	127037	82285
1y-W7	129735	112772	110155	93245	63660
1y-W8	135604	118005	115604	101180	70966
1y-W9	108024	90830	88689	72973	45546
3y-P1	175117	151591	150281	144644	119827
3y-P2	86577	77986	76163	67078	46116
3y-P3	93379	83577	81874	70665	53691
3y-P4	96159	85631	84602	81430	67417
3y-P5	127758	112302	110052	98322	63582
3y-P6	118888	103342	101650	94999	87845
3y-P7	87210	76327	75769	73977	72960
3y-P8	82701	71464	70191	63824	59017
3y-P9	68889	59598	57804	47520	35369
3y-W1	126028	111037	108986	95113	63442
3y-W2	265626	233501	229542	196477	107263
3y-W3	262972	232056	227999	192230	103748
3y-W4	291886	256315	252298	224978	157687
3y-W5	136968	117765	115051	97381	91805

Sample	Input	Filtered	Denoised	Merged	Non-chimeric
3y-W6	103602	89121	86570	72931	58371
3y-W7	102374	89286	87077	72625	53526
3y-W8	136077	119481	117552	109192	86935
3y-W9	123867	105459	103212	91362	76852
3y-W10	71486	61193	59788	53088	48132
3y-W11	95111	82229	80153	68823	52810
n1	86438	81787	79621	64490	50467
n2	84473	79468	77270	65432	53875
n3	87264	82327	80251	68386	54093
n4	81247	76746	75311	66698	56383
n5	81237	76800	74853	64083	51340
n6	81652	76401	74893	66328	57137
n7	82316	77499	75605	65031	52343
n8	81930	76474	73995	61452	49206
n9	83861	79318	77039	64085	49935
n10	84320	79744	78127	69536	58392
EP1	78204	73302	69599	58069	57009
EP2	67591	63447	60097	50763	49859
EP3	82398	77670	72914	51921	48643
EP4	84208	78786	74400	56613	54271
EP5	79870	75516	71273	53187	50892
EP6	84335	79473	75200	54564	51427
EP7	86268	80871	77530	66353	65200
EP8	78733	73852	70383	58493	57269
EP9	85362	80153	76819	67637	66782
EW1	80469	75519	72325	61646	60168
EW2	80504	75486	72350	58976	56550
EW3	83495	78579	75218	59592	56323
EW4	86848	81676	78330	62981	59512
EW5	85120	80177	76247	56441	51060
EW6	83042	77608	74198	53048	45811
EW7	72915	68183	66223	60476	59762
EW8	80083	74721	72423	65477	64672
EW9	86434	81675	78158	60004	56380
EW10	84137	79152	76009	61872	59135
EW11	82873	77857	75108	64162	61864

Table S3. Alpha diversity metrics

Sample	Observed_features	Chao1	faith_PD	Shannon	Simpson
2m-P1	1617	2356.351	107.558	7.423	0.973
2m-P2	1249	1943.275	87.961	7.099	0.966
2m-P3	1121	1744.981	83.528	6.724	0.967
2m-P4	1624	2696.964	111.482	7.874	0.986
2m-P5	1443	2254.313	101.652	7.148	0.956
2m-P6	1588	2416.683	109.722	7.744	0.986

Sample	Observed_features	Chao1	faith_PD	Shannon	Simpson
2m-P7	1791	3256.828	122.748	7.746	0.969
2m-P8	1191	2007.406	84.334	7.251	0.981
2m-P9	1611	2455.545	111.327	7.331	0.965
2m-P10	1473	2223.059	107.388	7.488	0.981
2m-P11	977	1478.396	76.403	7.248	0.979
2m-W1	1416	2148.013	99.222	8.009	0.989
2m-W2	1101	1426.837	67.144	7.351	0.970
2m-W3	1362	2095.373	92.793	7.387	0.971
2m-W4	1425	1947.828	97.786	7.006	0.952
2m-W5	1275	1868.555	87.754	7.516	0.978
2m-W6	1406	2182.588	91.679	7.793	0.981
2m-W7	1166	1744.258	78.935	6.474	0.904
2m-W8	1370	2115.728	93.795	7.318	0.968
2m-W9	918	1378.283	71.968	7.517	0.986
2m-W10	1054	1364.114	75.506	5.919	0.868
2m-W11	960	1209.712	65.748	5.927	0.919
1y-P1	880	1207.466	57.791	6.479	0.951
1y-P2	1169	1447.559	107.956	7.028	0.969
1y-P3	939	1735.470	75.088	7.431	0.988
1y-P4	1322	2402.000	92.337	8.160	0.992
1y-P5	914	1524.343	73.716	6.790	0.967
1y-P6	1322	2206.256	87.657	7.566	0.972
1y-P7	1118	1623.656	108.423	5.851	0.921
1y-P8	933	1450.471	63.855	7.174	0.980
1y-P9	836	1638.201	67.889	6.115	0.941
1y-P10	820	1266.298	69.500	6.930	0.981
1y-P11	760	994.164	62.379	6.289	0.909
1y-W1	814	1113.439	63.035	6.330	0.946
1y-W2	905	1386.169	57.054	5.650	0.867
1y-W3	3798	6753.249	268.960	7.526	0.892
1y-W4	1491	2948.658	93.726	7.359	0.979
1y-W5	2053	2324.915	163.844	9.923	0.995
1y-W6	1407	3060.344	92.825	7.237	0.965
1y-W7	1418	2802.360	96.901	7.354	0.966
1y-W8	1219	2269.006	84.610	7.417	0.982
1y-W9	1821	2970.933	98.257	7.883	0.977
3y-P1	846	1581.171	74.996	3.885	0.711
3y-P2	887	1080.460	68.960	4.976	0.836
3y-P3	1168	1671.453	76.907	5.199	0.832
3y-P4	501	711.690	50.253	3.541	0.746
3y-P5	1157	1943.819	87.171	6.487	0.960
3y-P6	635	881.584	58.370	5.565	0.894
3y-P7	438	534.207	46.970	3.775	0.712
3y-P8	652	779.735	56.289	6.441	0.956
3y-P9	1160	1606.952	82.792	7.308	0.974

Sample	Observed_features	Chao1	faith_PD	Shannon	Simpson
3y-W1	1377	1852.039	78.715	6.590	0.939
3y-W2	2160	4948.580	110.494	7.793	0.968
3y-W3	2016	4723.950	107.108	8.039	0.983
3y-W4	2045	4204.766	94.874	6.058	0.806
3y-W5	2063	2647.979	166.911	9.898	0.997
3y-W6	1354	1868.525	114.973	7.248	0.958
3y-W7	1207	1838.267	83.001	7.551	0.982
3y-W8	1340	2790.060	124.965	5.999	0.949
3y-W9	1321	2124.641	128.381	7.054	0.978
3y-W10	701	904.137	62.423	7.234	0.983
3y-W11	1177	1683.023	112.936	6.816	0.966
EP1	2856	3199.145	202.625	10.061	0.997
EP2	2974	3278.094	187.551	10.344	0.998
EP3	3107	3598.421	238.010	10.153	0.998
EP4	2989	3467.627	189.531	10.215	0.998
EP5	3246	3831.562	245.556	10.334	0.998
EP6	2958	3541.560	168.058	10.064	0.997
EP7	3051	3449.033	209.138	10.324	0.998
EP8	2955	3354.003	164.721	10.195	0.998
EP9	2970	3416.600	157.754	10.255	0.998
EW1	2376	2735.571	167.699	9.379	0.994
EW2	2688	3103.374	178.207	9.877	0.996
EW3	2709	3152.390	173.547	9.778	0.995
EW4	2921	3369.976	159.956	9.726	0.993
EW5	2793	3336.619	168.863	9.696	0.995
EW6	2703	3313.681	159.331	9.546	0.995
EW7	2350	2632.084	136.841	9.813	0.996
EW8	2582	2956.460	160.041	9.908	0.996
EW9	2784	3322.509	156.262	9.997	0.997
EW10	2585	3054.623	151.781	9.847	0.997
EW11	2392	2809.555	145.537	8.830	0.976
FP1	1016	1305.941	51.478	7.490	0.987
FP2	992	1317.094	49.617	7.056	0.955
FP3	972	1352.404	53.514	7.242	0.975
FP4	453	579.222	31.157	4.504	0.844
FP5	643	891.957	38.078	6.398	0.968
FP6	1112	1560.561	53.229	7.186	0.966
FP7	1170	1528.063	57.400	6.826	0.941
FP8	803	997.000	43.062	7.147	0.981
FW1	1333	1656.066	54.124	7.950	0.986
FW2	1586	2284.980	54.654	7.476	0.976
FW3	1214	1621.953	52.134	7.029	0.957
FW4	1831	2307.599	57.623	8.195	0.984
FW5	1133	1687.094	46.180	6.056	0.861
FW6	1306	1768.561	53.863	8.009	0.990

Sample	Observed_features	Chao1	faith_PD	Shannon	Simpson
FW7	1693	2402.539	49.880	7.290	0.947
FW8	715	916.718	32.771	5.076	0.881

Table S4. The results of LEfSe

Biomarker	Log value	Group	LDA	*P*-value	Class
c__Clostridia	5.49	P	-4.87	0.0135	13m
o__Lachnospirales	5.31	P	-4.83	0.0034	13m
f__Lachnospiraceae	5.31	P	-4.83	0.0034	13m
f__Erysipelatoclostridiaceae	4.74	P	-4.31	0.0088	13m
g__Coprobacillus	4.57	P	-4.16	0.0056	13m
g__Eisenbergiella	4.54	P	-4.09	0.0070	13m
g___Clostridium__innocuum	4.59	P	-3.98	0.0304	13m
g__Paludicola	2.78	P	-3.49	0.0413	13m
g__Blautia	4.01	P	-3.43	0.0070	13m
g__Eggerthella	3.68	P	-3.38	0.0205	13m
f__Eubacteriaceae	3.04	P	-3.34	0.0301	13m
g__Eubacterium	3.04	P	-3.34	0.0301	13m
f__Enterococcaceae	3.62	P	-3.27	0.0367	13m
g__Enterococcus	3.62	P	-3.27	0.0367	13m
g__RF39	3.58	P	-3.22	0.0367	13m
f__RF39	3.58	P	-3.22	0.0367	13m
o__RF39	3.58	P	-3.22	0.0367	13m
o__Aeromonadales	2.49	W	3.25	0.0206	13m
g__Aeromonas	2.49	W	3.26	0.0020	13m
f__Aeromonadaceae	2.49	W	3.26	0.0020	13m
g__Limnobacter	2.28	W	3.30	0.0168	13m
o__S085	2.26	W	3.43	0.0438	13m
g__S085	2.26	W	3.44	0.0438	13m
f__S085	2.26	W	3.47	0.0438	13m
g__Romboutsia	2.66	W	3.59	0.0060	13m
g__Desulfovibrio	5.16	P	-4.75	0.0053	2m
p__Desulfobacterota	5.26	P	-4.72	0.0235	2m
f__Desulfovibrionaceae	5.26	P	-4.72	0.0235	2m
c__Desulfovibrionia	5.26	P	-4.72	0.0235	2m
o__Desulfovibrionales	5.26	P	-4.72	0.0235	2m
g__Odoribacter	4.89	P	-4.21	0.0386	2m
f__Marinifilaceae	4.89	P	-4.21	0.0386	2m
f__Bifidobacteriaceae	2.25	P	-3.86	0.0004	2m
o__Bifidobacteriales	2.25	P	-3.84	0.0004	2m
g__Bifidobacterium	2.25	P	-3.84	0.0004	2m
g__Romboutsia	2.14	P	-3.71	0.0236	2m
f__Yersiniaceae	2.05	P	-3.59	0.0493	2m
g__Serratia	2.05	P	-3.58	0.0493	2m
g__Lachnospiraceae_UCG_004	2.91	P	-3.43	0.0235	2m

Biomarker	Log value	Group	LDA	*P*-value	Class
f__Lactobacillaceae	3.05	P	-3.23	0.0023	2m
g__Lactobacillus	3.05	P	-3.23	0.0023	2m
o__Enterobacterales	3.52	P	-3.21	0.0129	2m
g__Candidatus_Stoquefichus	3.75	W	3.43	0.0450	2m
g__Paraclostridium	4.24	W	4.01	0.0022	2m
o__Clostridiales	4.53	W	4.22	0.0003	2m
f__Clostridiaceae	4.53	W	4.22	0.0003	2m
g__Clostridium_sensu_stricto_1	4.53	W	4.22	0.0003	2m
g__Massilia	1.28	W	4.44	0.0318	2m
g__Roseburia	5.15	W	4.84	0.0241	2m
c__Clostridia	5.60	W	5.05	0.0004	2m
p__Firmicutes	5.68	W	5.13	0.0014	2m
f__Comamonadaceae	5.09	P	-4.85	0.0367	27m
g__Ralstonia	4.60	P	-4.36	0.0285	27m
f__Burkholderiaceae	4.60	P	-4.36	0.0150	27m
f__Chlamydiaceae	3.90	P	-3.67	0.0002	27m
o__Chlamydiales	3.90	P	-3.67	0.0002	27m
c__Chlamydiae	3.90	P	-3.67	0.0002	27m
g__Candidatus_Amphibiichlamydia	3.90	P	-3.67	0.0002	27m
g__Leuconostoc	1.52	P	-3.32	0.0438	27m
g__Roseomonas	1.73	P	-3.24	0.0438	27m
f__Nitrosomonadaceae	3.56	W	3.32	0.0247	27m
g__Dielma	3.77	W	3.37	0.0070	27m
f__Oscillospiraceae	3.85	W	3.41	0.0021	27m
g__Tannerellaceae	3.88	W	3.47	0.0016	27m
f__Ruminococcaceae	4.12	W	3.57	0.0135	27m
g__Odoribacter	4.28	W	3.87	0.0088	27m
g__Alistipes	4.30	W	3.88	0.0135	27m
f__Marinifilaceae	4.31	W	3.90	0.0070	27m
o__Oscillospirales	4.63	W	3.93	0.0304	27m
f__Rikenellaceae	4.36	W	3.94	0.0070	27m
o__Peptostreptococcales_Tissierellales	4.49	W	4.26	0.0441	27m
g__Parabacteroides	4.77	W	4.39	0.0021	27m
f__Tannerellaceae	4.82	W	4.44	0.0021	27m
g__Eisenbergiella	4.85	W	4.44	0.0088	27m
g__Bacteroides	5.24	W	4.81	0.0109	27m
f__Bacteroidaceae	5.24	W	4.81	0.0109	27m
o__Bacteroidales	5.46	W	5.04	0.0070	27m
c__Bacteroidia	5.47	W	5.05	0.0056	27m
p__Bacteroidota	5.47	W	5.05	0.0056	27m
o__Erysipelotrichales	5.21	FP	-4.84	0.0046	FMT
c__Bacilli	5.22	FP	-4.82	0.0209	FMT
c__Desulfovibrionia	4.99	FP	-4.62	0.0087	FMT
o__Desulfovibrionales	4.99	FP	-4.62	0.0087	FMT

Biomarker	Log value	Group	LDA	*P*-value	Class
f__Desulfovibrionaceae	4.99	FP	-4.62	0.0087	FMT
p__Desulfobacterota	4.99	FP	-4.62	0.0087	FMT
g__Desulfovibrio	4.94	FP	-4.57	0.0087	FMT
p__Verrucomicrobiota	4.48	FP	-4.15	0.0011	FMT
c__Chlamydiae	4.36	FP	-4.03	0.0011	FMT
o__Chlamydiales	4.36	FP	-4.03	0.0011	FMT
f__Chlamydiaceae	4.36	FP	-4.03	0.0011	FMT
g__Candidatus_Amphibiichlamydia	4.36	FP	-4.03	0.0011	FMT
f__Rikenellaceae	4.48	FP	-3.97	0.0460	FMT
c__Negativicutes	4.35	FP	-3.92	0.0357	FMT
o__Acidaminococcales	4.35	FP	-3.92	0.0357	FMT
g__Phascolarctobacterium	4.35	FP	-3.92	0.0357	FMT
f__Acidaminococcaceae	4.35	FP	-3.92	0.0357	FMT
f__Erysipelatoclostridiaceae	4.19	FP	-3.79	0.0016	FMT
g__Coprococcus	4.22	FP	-3.77	0.0011	FMT
o__Eubacteriales	2.28	FP	-3.72	0.0185	FMT
g__Blautia	4.06	FP	-3.70	0.0209	FMT
g___Clostridium__innocuum	3.95	FP	-3.68	0.0045	FMT
g__Bilophila	4.00	FP	-3.61	0.0085	FMT
f__Eubacteriaceae	2.28	FP	-3.54	0.0109	FMT
g__Eubacterium	2.28	FP	-3.53	0.0109	FMT
o__Burkholderiales	2.24	FP	-3.47	0.0249	FMT
g__Oscillibacter	3.84	FP	-3.45	0.0033	FMT
f__Oscillospiraceae	3.95	FP	-3.45	0.0357	FMT
g__Erysipelotrichaceae	3.74	FP	-3.45	0.0454	FMT
o__Gastranaerophilales	3.71	FP	-3.40	0.0056	FMT
f__Gastranaerophilales	3.71	FP	-3.40	0.0056	FMT
p__Cyanobacteria	3.71	FP	-3.40	0.0056	FMT
g__Gastranaerophilales	3.71	FP	-3.40	0.0056	FMT
c__Vampirivibrionia	3.71	FP	-3.40	0.0056	FMT
g__Hungatella	3.93	FP	-3.38	0.0274	FMT
f__Butyricicoccaceae	3.74	FP	-3.37	0.0023	FMT
g__Erysipelatoclostridium	3.62	FP	-3.34	0.0115	FMT
g__Butyricicoccus	3.66	FP	-3.33	0.0033	FMT
f__Muribaculaceae	2.71	FP	-3.26	0.0331	FMT
g__Muribaculaceae	2.71	FP	-3.25	0.0331	FMT
g__Anaerofilum	2.40	FP	-3.20	0.0256	FMT
g__Oxalobacter	1.91	FP	-3.20	0.0273	FMT
g__Lactobacillus	2.57	FW	3.29	0.0012	FMT
f__Lactobacillaceae	2.57	FW	3.29	0.0012	FMT
g__Ruminococcus	2.37	FW	3.55	0.0435	FMT
f__Bacteroidaceae	5.62	FW	4.85	0.0274	FMT
g__Bacteroides	5.62	FW	4.85	0.0274	FMT
p__Chloroflexi	4.85	EP	-4.20	0.0002	environment

Biomarker	Log value	Group	LDA	*P*-value	Class
c__Alphaproteobacteria	5.31	EP	-4.07	0.0034	environment
p__Acidobacteriota	4.65	EP	-4.05	0.0002	environment
c__Chloroflexia	4.57	EP	-3.89	0.0002	environment
o__Solirubrobacterales	4.83	EP	-3.89	0.0441	environment
c__Cyanobacteriia	4.29	EP	-3.88	0.0250	environment
o__Cyanobacteriales	4.20	EP	-3.85	0.0245	environment
c__Blastocatellia	4.34	EP	-3.77	0.0005	environment
f__Beijerinckiaceae	4.93	EP	-3.75	0.0070	environment
p__Gemmatimonadota	4.43	EP	-3.73	0.0034	environment
o__Rhizobiales	4.96	EP	-3.73	0.0167	environment
g__Microvirga	4.88	EP	-3.66	0.0205	environment
f__Rubrobacteriaceae	4.73	EP	-3.64	0.0367	environment
o__Rubrobacterales	4.73	EP	-3.64	0.0367	environment
c__Rubrobacteria	4.73	EP	-3.64	0.0367	environment
g__Rubrobacter	4.73	EP	-3.64	0.0367	environment
o__Thermomicrobiales	4.38	EP	-3.64	0.0002	environment
g__KD4_96	4.21	EP	-3.63	0.0003	environment
f__KD4_96	4.21	EP	-3.63	0.0003	environment
o__KD4_96	4.21	EP	-3.63	0.0003	environment
c__KD4_96	4.21	EP	-3.63	0.0003	environment
f__67_14	4.35	EP	-3.61	0.0034	environment
g__67_14	4.35	EP	-3.61	0.0034	environment
g__JG30_KF_CM45	4.33	EP	-3.60	0.0002	environment
f__JG30_KF_CM45	4.33	EP	-3.60	0.0002	environment
g__RB41	4.18	EP	-3.59	0.0002	environment
f__Pyrinomonadaceae	4.18	EP	-3.59	0.0002	environment
o__Pyrinomonadales	4.18	EP	-3.59	0.0002	environment
c__Acidimicrobiia	4.13	EP	-3.59	0.0002	environment
f__Phormidiaceae	3.92	EP	-3.59	0.0200	environment
f__Propionibacteriaceae	4.44	EP	-3.55	0.0012	environment
p__Myxococcota	4.08	EP	-3.51	0.0002	environment
f__Sphingomonadaceae	4.42	EP	-3.50	0.0027	environment
o__Sphingomonadales	4.42	EP	-3.50	0.0027	environment
f__Vicinamibacteraceae	4.06	EP	-3.50	0.0002	environment
c__Vicinamibacteria	4.06	EP	-3.50	0.0002	environment
o__Vicinamibacterales	4.06	EP	-3.50	0.0002	environment
g__Vicinamibacteraceae	4.02	EP	-3.46	0.0002	environment
c__Polyangia	3.94	EP	-3.46	0.0002	environment
g__Friedmanniella	4.32	EP	-3.45	0.0010	environment
o__Microtrichales	3.97	EP	-3.44	0.0002	environment
g__Sphingomonas	4.31	EP	-3.43	0.0021	environment
g__Tychonema_CCAP_1459_11B	3.79	EP	-3.41	0.0063	environment
o__Acetobacterales	4.36	EP	-3.39	0.0056	environment
f__Acetobacteraceae	4.36	EP	-3.39	0.0056	environment

Biomarker	Log value	Group	LDA	*P*-value	Class
o__Gemmatimonadales	4.09	EP	-3.39	0.0012	environment
c__Gemmatimonadetes	4.09	EP	-3.39	0.0012	environment
f__Gemmatimonadaceae	4.09	EP	-3.39	0.0012	environment
c__Acidobacteriae	4.05	EP	-3.38	0.0003	environment
o__Kallotenuales	3.95	EP	-3.38	0.0004	environment
g__AKIW781	3.95	EP	-3.38	0.0004	environment
f__AKIW781	3.95	EP	-3.38	0.0004	environment
f__Bryobacteraceae	4.05	EP	-3.37	0.0003	environment
o__Bryobacterales	4.05	EP	-3.37	0.0003	environment
g__Bryobacter	4.05	EP	-3.37	0.0003	environment
g__Craurococcus_Caldovatus	4.16	EP	-3.35	0.0044	environment
f__Ilumatobacteraceae	3.80	EP	-3.31	0.0003	environment
p__Nitrospirota	3.86	EP	-3.30	0.0002	environment
o__Nitrospirales	3.86	EP	-3.30	0.0002	environment
c__Nitrospiria	3.86	EP	-3.30	0.0002	environment
f__Nitrospiraceae	3.86	EP	-3.30	0.0002	environment
g__Nitrospira	3.86	EP	-3.30	0.0002	environment
g__Solirubrobacteraceae	3.83	EP	-3.29	0.0002	environment
g__CL500_29_marine	3.77	EP	-3.26	0.0005	environment
p__Verrucomicrobiota	3.79	EP	-3.25	0.0002	environment
c__Verrucomicrobiae	3.77	EP	-3.23	0.0002	environment
g__Roseisolibacter	4.00	EP	-3.23	0.0016	environment
g__Candidatus_Alysiosphaera	3.96	EP	-3.22	0.0004	environment
o__Tistrellales	4.02	EP	-3.21	0.0005	environment
f__Geminicoccaceae	4.02	EP	-3.21	0.0005	environment
o__Blastocatellales	3.76	EP	-3.21	0.0016	environment
f__Blastocatellaceae	3.76	EP	-3.21	0.0016	environment
g__Arthrobacter	3.72	EW	3.35	0.0003	environment
g__Modestobacter	4.40	EW	3.37	0.0205	environment
g__Streptomyces	4.07	EW	3.40	0.0367	environment
g__Microbacterium	3.94	EW	3.41	0.0034	environment
g__Kocuria	3.85	EW	3.42	0.0002	environment
g__Bacillus	4.21	EW	3.42	0.0034	environment
f__Bacillaceae	4.22	EW	3.45	0.0044	environment
f__Microbacteriaceae	4.20	EW	3.60	0.0021	environment
g__Planococcus	3.98	EW	3.67	0.0001	environment
g__Planomicrobium	4.03	EW	3.70	0.0002	environment
g__Adhaeribacter	4.25	EW	3.72	0.0016	environment
g__Cellulomonas	4.50	EW	3.85	0.0021	environment
o__Cytophagales	4.50	EW	3.85	0.0034	environment
f__Hymenobacteraceae	4.40	EW	3.85	0.0016	environment
f__Cellulomonadaceae	4.50	EW	3.85	0.0016	environment
f__Planococcaceae	4.37	EW	4.01	0.0002	environment
o__Bacillales	4.60	EW	4.12	0.0005	environment

Biomarker	Log value	Group	LDA	*P*-value	Class
p__Firmicutes	4.68	EW	4.16	0.0003	environment
c__Bacilli	4.68	EW	4.16	0.0003	environment
c__Gammaproteobacteria	4.89	EW	4.22	0.0056	environment
o__Burkholderiales	4.85	EW	4.23	0.0034	environment
g__Pseudarthrobacter	4.91	EW	4.28	0.0002	environment
g__Massilia	4.71	EW	4.32	0.0002	environment
f__Oxalobacteraceae	4.75	EW	4.32	0.0002	environment
p__Actinobacteriota	5.72	EW	4.37	0.0441	environment
f__Micrococcaceae	4.98	EW	4.39	0.0002	environment
o__Micrococcales	5.17	EW	4.56	0.0002	environment
c__Actinobacteria	5.61	EW	4.60	0.0034	environment

Table S5. The results of differential KEGG pathway analysis

Pathway	LogFC	SE	*P*-values	Description	Class
ko00020	-0.16	0.14	0.0090	Citrate cycle (TCA cycle)	2m
ko00072	0.19	0.21	0.0300	Synthesis and degradation of ketone bodies	2m
ko00130	-0.42	0.21	0.0000	Ubiquinone and other terpenoid-quinone biosynthesis	2m
ko00140	-0.31	0.43	0.0490	Steroid hormone biosynthesis	2m
ko00253	-0.68	0.98	0.0100	Tetracycline biosynthesis	2m
ko00280	-0.11	0.12	0.0330	Valine, leucine and isoleucine degradation	2m
ko00281	-0.33	0.33	0.0120	Geraniol degradation	2m
ko00350	-0.09	0.08	0.0100	Tyrosine metabolism	2m
ko00360	-0.14	0.11	0.0020	Phenylalanine metabolism	2m
ko00361	0.23	0.27	0.0420	Chlorocyclohexane and chlorobenzene degradation	2m
ko00363	-1.20	0.53	0.0043	Bisphenol degradation	2m
ko00460	4.47	2.19	0.0000	Cyanoamino acid metabolism	2m
ko00510	-0.29	0.20	0.0000	N-Glycan biosynthesis	2m
ko00523	0.31	1.11	0.0360	Polyketide sugar unit biosynthesis	2m
ko00531	-0.30	0.38	0.0410	Glycosaminoglycan degradation	2m
ko00540	-0.38	0.28	0.0010	Lipopolysaccharide biosynthesis	2m
ko00561	0.12	0.11	0.0080	Glycerolipid metabolism	2m
ko00562	-0.18	0.13	0.0030	Inositol phosphate metabolism	2m
ko00591	0.17	0.17	0.0230	Linoleic acid metabolism	2m
ko00601	-0.02	0.02	0.0000	Glycosphingolipid biosynthesis - lacto and neolacto series	2m
ko00720	-0.12	0.11	0.0210	Carbon fixation pathways in prokaryotes	2m
ko00780	-0.13	0.13	0.0250	Biotin metabolism	2m
ko00785	-0.30	0.18	0.0000	Lipoic acid metabolism	2m
ko00830	-0.92	0.41	0.0100	Retinol metabolism	2m
ko00903	-2.10	1.48	0.0209	Limonene and pinene degradation	2m
ko00920	-0.10	0.10	0.0200	Sulfur metabolism	2m
ko00930	-0.53	0.66	0.0350	Caprolactam degradation	2m
ko00941	-0.96	0.92	0.0060	Flavonoid biosynthesis	2m
ko01053	-0.54	0.44	0.0060	Biosynthesis of siderophore group nonribosomal peptides	2m
ko03015	-1.34	0.84	0.0172	mRNA surveillance pathway	2m

Pathway	LogFC	SE	*P*-values	Description	Class
ko04141	-0.18	0.14	0.0040	Protein processing in endoplasmic reticulum	2m
ko04146	-0.15	0.16	0.0310	Peroxisome	2m
ko04210	-0.40	0.37	0.0070	Apoptosis	2m
ko04962	-1.52	0.76	0.0153	Vasopressin-regulated water reabsorption	2m
ko04974	-0.32	0.37	0.0230	Protein digestion and absorption	2m
ko05110	-2.19	1.17	0.0000	Vibrio cholerae infection	2m
ko05131	-2.69	1.00	0.0143	Shigellosis	2m
ko05143	-0.58	0.69	0.0210	African trypanosomiasis	2m
ko05145	-2.59	0.80	0.0000	Toxoplasmosis	2m
ko05146	-0.30	0.27	0.0040	Amoebiasis	2m
ko05150	0.64	0.70	0.0100	Staphylococcus aureus infection	2m
ko00010	-0.17	0.19	0.0260	Glycolysis / Gluconeogenesis	13m
ko00040	-0.17	0.14	0.0030	Pentose and glucuronate interconversions	13m
ko00051	-0.27	0.31	0.0450	Fructose and mannose metabolism	13m
ko00052	-0.27	0.20	0.0030	Galactose metabolism	13m
ko00196	2.30	1.15	0.0225	Photosynthesis - antenna proteins	13m
ko00400	-0.12	0.14	0.0480	Phenylalanine, tyrosine and tryptophan biosynthesis	13m
ko00472	-0.77	0.63	0.0080	D-Arginine and D-ornithine metabolism	13m
ko00500	-0.21	0.17	0.0030	Starch and sucrose metabolism	13m
ko00520	-0.19	0.22	0.0410	Amino sugar and nucleotide sugar metabolism	13m
ko00521	-0.12	0.14	0.0410	Streptomycin biosynthesis	13m
ko00561	-0.16	0.17	0.0310	Glycerolipid metabolism	13m
ko00633	0.23	0.23	0.0310	Nitrotoluene degradation	13m
ko00730	-0.18	0.15	0.0090	Thiamine metabolism	13m
ko00910	-0.09	0.10	0.0200	Nitrogen metabolism	13m
ko00983	-0.13	0.16	0.0460	Drug metabolism - other enzymes	13m
ko01056	3.03	0.89	0.0031	Biosynthesis of type II polyketide backbone	13m
ko03013	-0.14	0.17	0.0500	RNA transport	13m
ko04144	1.73	1.13	0.0176	Endocytosis	13m
ko04512	1.42	0.72	0.0280	ECM-receptor interaction	13m
ko04621	-0.16	0.18	0.0460	NOD-like receptor signaling pathway	13m
ko04910	-0.18	0.21	0.0430	Insulin signaling pathway	13m
ko00020	0.22	0.15	0.0010	Citrate cycle (TCA cycle)	27m
ko00040	0.26	0.19	0.0080	Pentose and glucuronate interconversions	27m
ko00312	0.67	0.73	0.0330	beta-Lactam resistance	27m
ko00350	-0.16	0.18	0.0290	Tyrosine metabolism	27m
ko00361	-0.69	0.61	0.0130	Chlorocyclohexane and chlorobenzene degradation	27m
ko00380	-0.29	0.33	0.0490	Tryptophan metabolism	27m
ko00401	1.53	1.74	0.0000	Novobiocin biosynthesis	27m
ko00510	0.97	0.70	0.0010	N-Glycan biosynthesis	27m
ko00511	0.65	0.59	0.0140	Other glycan degradation	27m
ko00540	0.42	0.48	0.0370	Lipopolysaccharide biosynthesis	27m
ko00562	0.15	0.18	0.0400	Inositol phosphate metabolism	27m
ko00591	-0.35	0.34	0.0190	Linoleic acid metabolism	27m

Pathway	LogFC	SE	*P*-values	Description	Class
ko00600	0.52	0.53	0.0310	Sphingolipid metabolism	27m
ko00625	-0.21	0.23	0.0320	Chloroalkane and chloroalkene degradation	27m
ko00627	-0.35	0.36	0.0380	Aminobenzoate degradation	27m
ko00643	-0.68	0.62	0.0190	Styrene degradation	27m
ko00750	0.24	0.23	0.0280	Vitamin B6 metabolism	27m
ko00780	0.23	0.21	0.0180	Biotin metabolism	27m
ko00790	0.26	0.19	0.0040	Folate biosynthesis	27m
ko00830	0.79	0.47	0.0452	Retinol metabolism	27m
ko00860	0.26	0.25	0.0310	Porphyrin and chlorophyll metabolism	27m
ko00903	-0.82	0.35	0.0119	Limonene and pinene degradation	27m
ko00906	-1.12	1.01	0.0110	Carotenoid biosynthesis	27m
ko00943	-1.65	2.37	0.0460	Isoflavonoid biosynthesis	27m
ko01053	0.63	0.73	0.0340	Biosynthesis of siderophore group nonribosomal peptides	27m
ko03015	1.41	0.70	0.0180	mRNA surveillance pathway	27m
ko04146	0.21	0.16	0.0050	Peroxisome	27m
ko04974	0.75	0.94	0.0470	Protein digestion and absorption	27m
ko05010	-1.71	0.67	0.0000	Alzheimer's disease	27m
ko05111	-0.20	0.21	0.0230	Vibrio cholerae pathogenic cycle	27m
ko05146	0.39	0.44	0.0360	Amoebiasis	27m
ko05410	0.96	0.58	0.0433	Hypertrophic cardiomyopathy (HCM)	27m

Table S6. qRT-PCR primer sequences

Gene	Forward Primer	Reverse Primer	Sequence Source
IL-1β	GGGCGCTTCCTCTTCTACAG	TGTCCAAAGGCACATGTAAGGA	under review
IFN-β	TCCTCAACAGCAAGATGGGAT	TGGTGGTGTGGTTTTGTTGGAAG	under review
MYD88	TGCCCGAACGACATTCACTT	TGGTGATGGACCAGACACAC	XM_035130082.1
STAT3	CACCAACATCCTGGTCTCCC	ACTACCTGGGTCAGTGGCTT	XM_035136516.1
TNFα	TGTGGCACCCTCCATACTTG	GCAGGTGTCGCTATGGAACA	under review
TRIF	ACGGAGAACTTCAAGAGCCG	CTTTGGCAATGAAGGGCACC	XM_035118973.1
EF1A1	GGATGGAAAGTGACCCGTAAG	ACATCCTGAAGTGGCAGACGC	XM_028722768.1

Table S7. Quantitative analysis of short chain fatty acids

Sample	Acetate	Propionate	Butyrate	Isobutyrate	Valerate	Isovalerate
3y-P1	0.2292	0.0667	0.0337	0.0038	0.0059	0.0051
3y-P2	0.5745	0.2243	0.1814	0.0098	0.0129	0.0073
3y-P3	0.4630	0.1804	0.1478	0.0081	0.0105	0.0059
3y-P4	0.2269	0.0304	0.0796	0.0049	0.0046	0.0034
3y-P5	0.2985	0.1180	0.0499	0.0092	0.0089	0.0080
3y-P6	0.1932	0.0650	0.0597	0.0038	0.0059	0.0045
3y-P7	0.5765	0.1221	0.1559	0.0067	0.0087	0.0061
3y-P8	0.1575	0.0725	0.0360	0.0040	0.0040	0.0038
3y-W1	0.8610	0.1795	0.2858	0.0092	0.0110	0.0050
3y-W2	0.7389	0.5555	0.3715	0.0180	0.0333	0.0187
3y-W3	1.0645	0.1654	0.4507	0.0130	0.0327	0.0067

Sample	Acetate	Propionate	Butyrate	Isobutyrate	Valerate	Isovalerate
3y-W4	0.6391	0.4609	0.5407	0.0278	0.0392	0.0376
3y-W5	0.2767	0.1250	0.0563	0.0061	0.0134	0.0060
3y-W6	0.2210	0.1282	0.0535	0.0055	0.0101	0.0051
3y-W7	0.6528	0.1729	0.2804	0.0119	0.0134	0.0063
3y-W8	0.7394	0.4536	0.2213	0.0259	0.0195	0.0109
FP1	0.4697	0.0676	0.0821	0.0054	0.0071	0.0048
FP2	0.2632	0.1168	0.0742	0.0088	0.0119	0.0060
FP3	0.3290	0.0917	0.1827	0.0055	0.0121	0.0047
FP4	0.2896	0.1091	0.1739	0.0054	0.0101	0.0071
FP5	0.1982	0.0544	0.0810	0.0055	0.0112	0.0060
FP6	0.3414	0.0735	0.1166	0.0041	0.0086	0.0055
FP7	0.0738	0.0177	0.0509	0.0035	0.0046	0.0034
FP8	0.0746	0.0200	0.0290	0.0034	0.0043	0.0034
FW1	0.9946	0.1991	0.3514	0.0147	0.0113	0.0071
FW2	0.3347	0.0906	0.1327	0.0065	0.0113	0.0067
FW3	0.6208	0.2080	0.1171	0.0136	0.0126	0.0100
FW4	0.4645	0.1426	0.1071	0.0110	0.0161	0.0098
FW5	0.3210	0.1010	0.1287	0.0123	0.0054	0.0060
FW6	0.5427	0.1192	0.3251	0.0074	0.0142	0.0065
FW7	0.7335	0.1808	0.2378	0.0117	0.0104	0.0073
FW8	0.7322	0.1790	0.2698	0.0208	0.0207	0.0133

Table S8. Sample information for fecal microbiota transplantation (FMT) experiments

Sample	Sex	Body Mass	Snout-vent Length	Class
Present climate 1	M	8.620	69.07	Donors
Present climate 2	M	7.745	63.03	Donors
Present climate 3	M	8.428	68.39	Donors
Warming climate 1	M	10.512	70.59	Donors
Warming climate 2	M	10.142	68.85	Donors
Warming climate 3	M	8.624	67.91	Donors
Warming climate 4	M	7.367	65.43	Donors
Warming climate 5	M	6.319	66.80	Donors
FP1	M	6.677	61.43	Recipients
FP2	M	6.452	64.08	Recipients
FP3	M	6.314	63.23	Recipients
FP4	M	5.293	59.11	Recipients
FP5	M	6.65	59.22	Recipients
FP6	M	5.583	62.96	Recipients
FP7	M	7.031	66.47	Recipients
FP8	M	6.417	65.25	Recipients
FW1	M	6.201	66.29	Recipients
FW2	M	6.77	64.86	Recipients
FW3	M	6.046	62.82	Recipients
FW4	M	6.15	63.02	Recipients
FW5	M	5.474	62.26	Recipients

Sample	Sex	Body Mass	Snout-vent Length	Class
FW6	M	6.658	62.96	Recipients
FW7	M	6.046	62.64	Recipients
FW8	M	5.776	60.28	Recipients

Table S9. Statistics of 16S rRNA gene sequencing data for fecal microbiota transplantation (FMT) experiments

Sample	Input	Filtered	Denoised	Merged	Non-chimeric
FP1	73536	69338	68049	59605	43461
FP2	76942	72659	71260	63026	51954
FP3	77326	73377	71722	62132	50617
FP4	81540	77498	76805	73381	60309
FP5	77560	73549	72575	67316	58628
FP6	77298	72574	71090	62284	47963
FP7	65105	61548	60081	51159	37269
FP8	72845	68989	67926	62107	55223
FW1	73916	70325	68361	54977	38837
FW2	85949	78804	77389	68062	44493
FW3	73569	67649	66068	56242	40215
FW4	74010	67919	66315	53903	32048
FW5	106560	98700	97282	88726	68068
FW6	80535	74311	72619	61981	40887
FW7	84050	76640	75203	65615	43668
FW8	73050	67451	66487	61908	50271
